# Unsupervised and supervised discovery of tissue cellular neighborhoods from cell phenotypes

**DOI:** 10.1038/s41592-023-02124-2

**Published:** 2024-01-08

**Authors:** Yuxuan Hu, Jiazhen Rong, Yafei Xu, Runzhi Xie, Jacqueline Peng, Lin Gao, Kai Tan

**Affiliations:** 1 School of Computer Science and Technology, Xidian University, Xi’an, China; 2 Graduate Group in Genomics and Computational Biology, Perelman School of Medicine, University of Pennsylvania, Philadelphia, PA USA; 3 Division of Oncology and Center for Childhood Cancer Research, Children’s Hospital of Philadelphia, Philadelphia, PA USA; 4 Department of Pediatrics, Perelman School of Medicine, University of Pennsylvania, Philadelphia, PA USA

**Keywords:** Computational models, Image processing

## Abstract

It is poorly understood how different cells in a tissue organize themselves to support tissue functions. We describe the CytoCommunity algorithm for the identification of tissue cellular neighborhoods (TCNs) based on cell phenotypes and their spatial distributions. CytoCommunity learns a mapping directly from the cell phenotype space to the TCN space using a graph neural network model without intermediate clustering of cell embeddings. By leveraging graph pooling, CytoCommunity enables de novo identification of condition-specific and predictive TCNs under the supervision of sample labels. Using several types of spatial omics data, we demonstrate that CytoCommunity can identify TCNs of variable sizes with substantial improvement over existing methods. By analyzing risk-stratified colorectal and breast cancer data, CytoCommunity revealed new granulocyte-enriched and cancer-associated fibroblast-enriched TCNs specific to high-risk tumors and altered interactions between neoplastic and immune or stromal cells within and between TCNs. CytoCommunity can perform unsupervised and supervised analyses of spatial omics maps and enable the discovery of condition-specific cell–cell communication patterns across spatial scales.

## Abstract (web summary)

CytoCommunity enables both supervised and unsupervised analyses of spatial omics data in order to identify complex tissue cellular neighborhoods based on cell phenotypes and spatial distributions.

## Main

To understand the structure-function relationship of a tissue, the concept of tissue cellular neighborhoods (TCNs) or spatial domains has been proposed as a recurrent functional unit in which different cell types organize themselves to support tissue functions^1–3^. With the development of spatial omics, there is a critical need for computational methods^2,4–10^ for identifying spatial domains in tissues. Several pioneering methods have been developed, which can be roughly classified into non-deep-learning-based and deep-learning-based approaches. As representative of the first category, Giotto^2,4^ and BayesSpace^5^ identify spatial domains with similar gene expression patterns based on probabilistic graphical models and spatial transcriptomics data. Spatial-LDA^9^ uses the latent Dirichlet allocation topic model to identify spatially coherent patterns based on cell-type counts and cell spatial coordinates. UTAG^10^ uses message passing to combine cell molecular features and spatial location information followed by clustering to identify spatial domains. As a deep-learning-based method, stLearn^6^ uses a convolutional neural network model to extract features from a histological image and measures morphological similarity between neighboring cells or spots in spatial transcriptomics data to smooth gene expression. Clustering is then performed on the normalized expression data for spatial domain identification. SpaGCN^7^, STAGATE^8^ and SPACE-GM^11^ first use graph neural network (GNN) models to integrate gene expression or cell-type information and spatial location data to generate embedding representations of cells or spots and then perform clustering on those embeddings to identify spatial domains.

Of the existing methods, several (BayesSpace, stLearn, SpaGCN and STAGATE) were originally designed for spatial transcriptomics data and thus use the expression of hundreds or thousands of genes as features to infer TCNs. Such methods may not be applicable to spatial proteomics data^3,12^ that only have a few tens of protein expression features available. Additionally, using gene expression data as input cannot directly establish the relationship between cell types and TCNs in a tissue, making the interpretation of TCNs challenging. Given a cohort of tissue samples associated with different conditions (for example, disease risk and patient prognosis), it is important to identify condition-specific TCNs with more biological and clinical relevance. A representative condition-specific TCN in cancer tissues is the tertiary lymphoid structure, which is typically present in low-risk but not in high-risk patients with many cancer types^13^. Most of the existing methods are designed to detect TCNs in individual tissue samples using unsupervised learning and thus are not applicable for the de novo identification of condition-specific TCNs. SPACE-GM can generate cell embedding features using supervised learning. Subsequently, TCNs are identified using unsupervised clustering on these embeddings. To our knowledge, no method currently enables both unsupervised TCN detection in individual tissue maps and de novo identification of condition-specific TCNs using supervised learning and tissue sample labels explicitly.

In this study, we describe the CytoCommunity algorithm used to identify TCNs that can be applied in either an unsupervised or supervised fashion. We formulate TCN identification as a community detection problem on graphs and use a graph minimum cut (MinCut)-based GNN model to identify TCNs. CytoCommunity directly uses cell phenotypes as features to learn TCN partitions and thus facilitates the interpretation of TCN functions. CytoCommunity can also identify condition-specific TCNs from a cohort of labeled tissue samples by leveraging differentiable graph pooling and sample labels, which is an effective strategy to address the difficulty of graph alignment. Our GNN framework directly learns TCN partitions and is thus different from SpaGCN^7^, STAGATE^8^ and SPACE-GM^11^, which use clustering of cell embeddings after the GNN step to identify spatial domains. Consequently, the resulting spatial domains identified by these methods are dependent not only on the GNN models but also on secondary clustering algorithms. Moreover, the intermediate clustering step makes it difficult to adopt a supervised learning framework to find condition-specific TCNs.

Using diverse types of single-cell and spot resolution spatial omics datasets, we benchmarked the performance of unsupervised CytoCommunity on the detection of TCNs of variable sizes in individual tissue samples and supervised CytoCommunity on the identification of condition-specific TCNs in tumor tissue samples. We also demonstrated the ability of supervised CytoCommunity to reveal changes within and between TCN communication in the tumor tissues of patients with different risks and prognoses.

## Results

### Overview of CytoCommunity

**Fig. 1:**
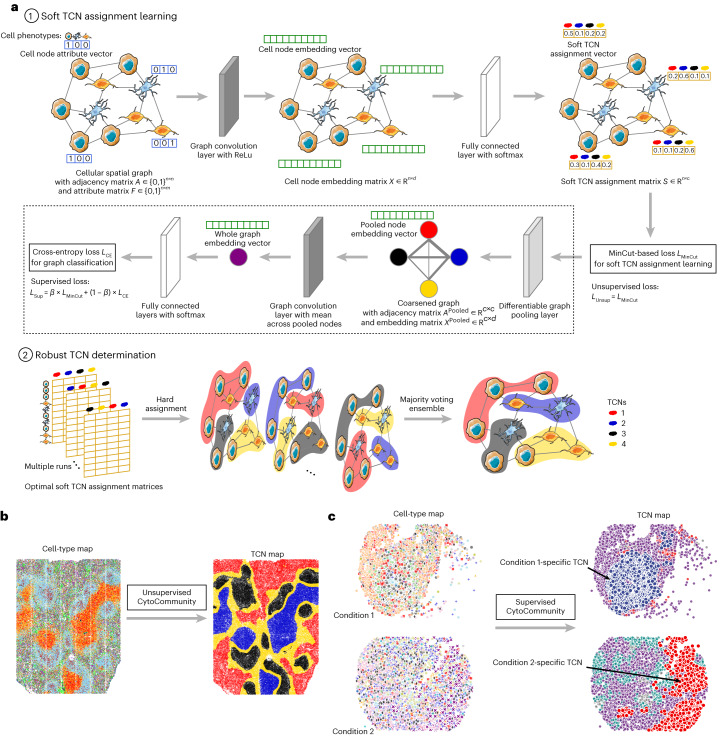
Schematic diagram of the CytoCommunity algorithm. Given single-cell spatial maps with cell phenotype annotation and cell spatial coordinates, TCN identification is formulated as a community detection problem on graphs. **a**, The algorithm includes a soft TCN assignment module and a TCN ensemble module. First, a *k*-NN-based cellular spatial graph is constructed using cell spatial coordinates. Each node represents a cell and its *m*-dimensional attribute vector (blue) encodes the cell phenotype. *m*, number of cell phenotypes; *n*, number of cells. A basic GNN is applied to this cellular spatial graph to obtain a *d*-dimensional embedding vector (green) for each node. Embedding dimensions are specified according to users. A fully connected neural network is used to transform cell node embeddings to soft TCN assignments (yellow vectors) of nodes, representing the probabilities of cells belonging to *c* TCNs. The number of TCNs are specified according to users. The graph MinCut-based loss function (*L*_MinCut_) is used to learn the optimal soft TCN assignments of all nodes. This loss function can be used alone for an unsupervised learning task. In a supervised learning task, differentiable graph pooling, graph convolution and two fully connected layers with the cross-entropy loss function *L*_CE_ (for sample classification, bordered by a dashed rectangular box) are added on top of the soft TCN assignment module. The overall supervised loss function is a linear combination of *L*_MinCut_ and *L*_CE_ with a weight parameter *β*. In the TCN ensemble module, the first module can be run multiple times to generate multiple optimal soft TCN assignment matrices. Hard assignment is conducted for each of them and an ensemble procedure is performed on those hard TCN assignments using a majority vote strategy to determine the final robust TCNs. **b**, For an unsupervised learning task, CytoCommunity identified TCNs for each tissue sample individually. **c**, For a supervised learning task, using a dataset of tissue samples associated with different conditions as the input, CytoCommunity enabled de novo identification of condition-specific TCNs under the supervision of sample labels.

CytoCommunity consists of two components: a GNN-based soft TCN assignment module and a TCN ensemble module to determine a robust set of TCNs (Fig. 1a). CytoCommunity can be used for either unsupervised (Fig. 1b and Methods) or supervised (Fig. 1c and Methods) learning tasks. For unsupervised learning, a MinCut-based loss function^14^ is used alone to detect TCNs in individual single-cell spatial maps without any sample labels. In a supervised learning task for de novo identification of condition-specific TCNs, the overall loss function is a linear combination of the MinCut-based loss function and a cross-entropy loss function that is used for sample classification. To alleviate the instability of graph partitioning based on GNN, a majority-vote-based ensemble procedure is performed on multiple optimal soft TCN assignment matrices generated by the first GNN module to determine the final robust set of TCNs.

**Extended Data Fig. 1:**
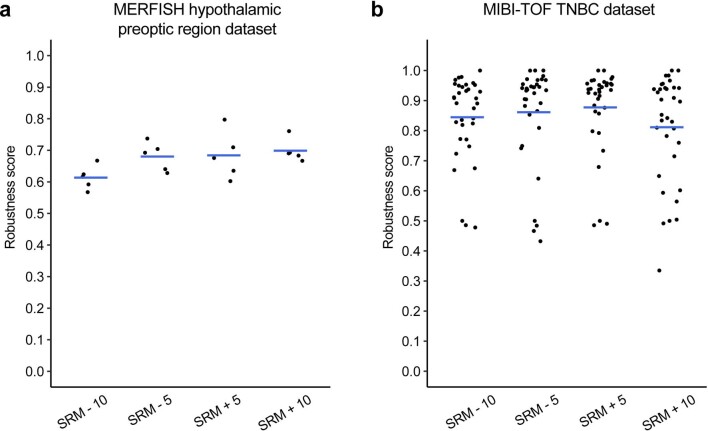
Robustness assessment of CytoCommunity performance using different values of K in K-nearest-neighbor graphs. **(a)** Assessment using the mouse hypothalamic preoptic region MERFISH dataset. **(b)** Assessment using the human triple-negative breast cancer (TNBC) MIBI-TOF dataset. Robustness score is defined as the average Jaccard index between original TCN partitions and new TCN partitions generated using different K values. SRM, square root of the average number of cells (SRM) across images in the dataset. K values were varied around the SRM values. Each black point represents the robustness performance on a given single-cell spatial image (n = 5 for the MERFISH dataset and n = 34 for the MIBI-TOF dataset). Blue horizontal bars represent the means of each group.

**Extended Data Fig. 2:**
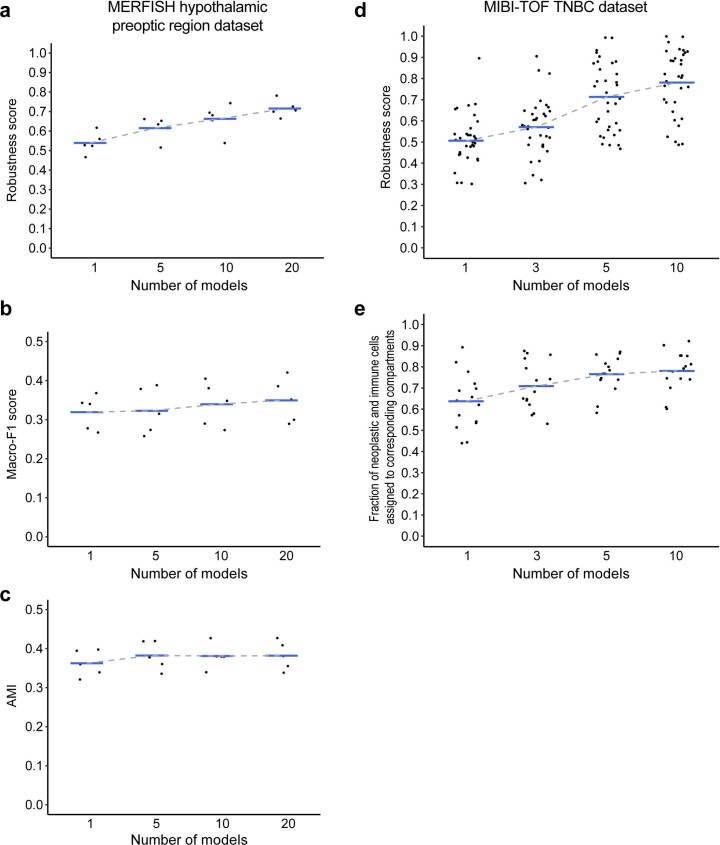
Effect of number of models of the ensemble procedure on the performance of CytoCommunity. **(a-c)** Unsupervised CytoCommunity was applied to the mouse hypothalamic preoptic region MERFISH dataset using different number of models learned from the soft TCN assignment module. The effect of number of models on the robustness **(a)** and accuracy including Macro-F1 score **(b)** and adjusted mutual information (AMI) score **(c)** was assessed. Macro-F1 score and AMI score were computed by comparing TCN partitions generated using different number of models with manually annotated hypothalamic nuclei (Fig. 3b). Robustness score was computed as the average Jaccard index between those TCN partitions and new TCN partitions generated by additional three replicated experiments. Each black point represents the robustness or the accuracy performance on a given MERFISH image (n = 5). **(d, e)** Supervised CytoCommunity was applied to the human TNBC MIBI-TOF dataset using different number of models trained based on a 10-fold cross-validation. The effect of number of models on the robustness **(d)** and accuracy **(e)** was assessed. Robustness score was computed the same as before and each black point represents the robustness performance on a given compartmentalized or mixed tumor image (n = 34). Accuracy performance was evaluated using the fractions of neoplastic and immune cells correctly assigned to the neoplastic- and immune-dominated TCNs. Each black point represents the accuracy performance on a given compartmentalized tumor image (n = 15). For all panels, blue horizontal bars represent the means of each group with grey dashed lines connecting them.

**Extended Data Fig. 3:**
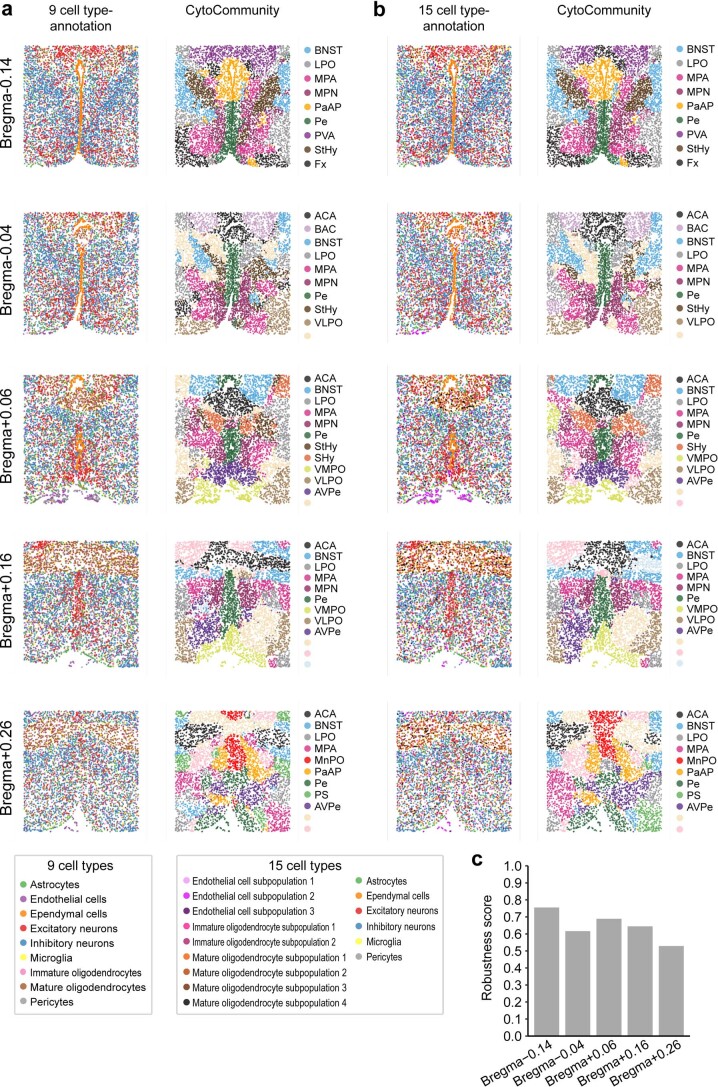
Robustness assessment of CytoCommunity performance using different granularity of annotated cell types. Mouse hypothalamic preoptic region MERFISH dataset was used due to the complex cell types in this tissue. **(a)** Cell types and TCNs identified using 9 coarse-grained cell type annotations as the input. **(b)** Cell types and TCNs identified using 15 fine-grained cell type annotations as the input. Both sets of cell type annotations were downloaded from the original study^17^. **(c)** Robustness score is defined as the average Jaccard index between the two sets of TCNs generated using the two sets of cell type annotations.

**Extended Data Fig. 4:**
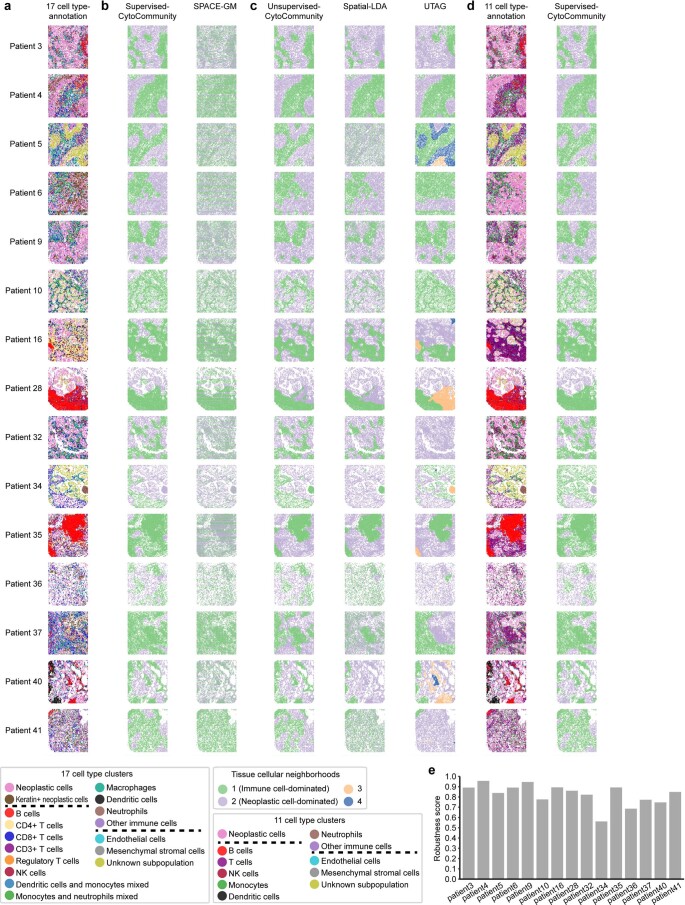
Performance evaluation using MIBI-TOF data of compartmentalized tumors from triple-negative breast cancer patients. 15 single-cell-resolution images of compartmentalized tumors generated using the MIBI-TOF technology. **(a)** Cells are colored based on 17 cell types annotated by the original study^19^. **(b-c)** TCNs identified by supervised **(b)** and unsupervised methods **(c)**. **(d)** Left, cells are colored based on 11 cell types by combining similar cell types annotated by the original study into a single major cell type. Specifically, two neoplastic cell types are combined into one neoplastic cell type. Four T cell subtypes are combined into a single T cell cluster. Two mixed cell type clusters and macrophages are combined into a single monocyte cluster. Right, TCNs identified by CytoCommunity. **(e)** Robustness score is defined as the average Jaccard index between the two sets of TCNs generated using the two sets of cell type annotations.

**Extended Data Fig. 5:**
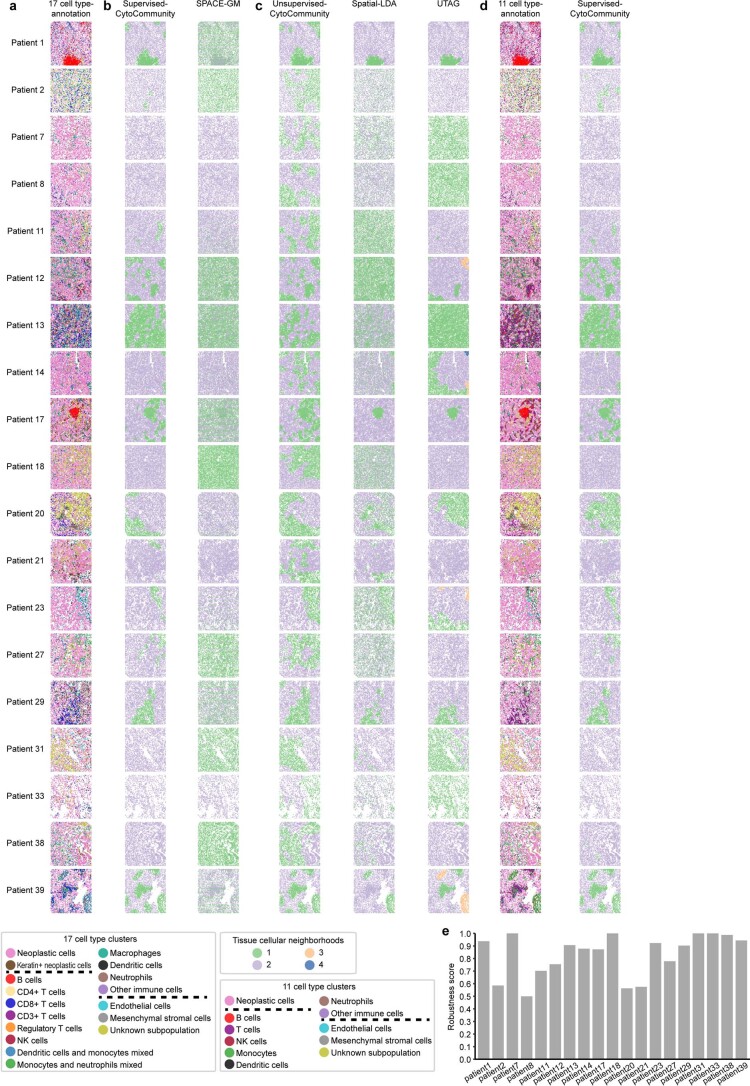
Performance evaluation using MIBI-TOF data of mixed tumors from triple-negative breast cancer patients. 19 single-cell-resolution images of mixed tumors generated using the MIBI-TOF technology. **(a)** Cells are colored based on 17 cell types annotated by the original study^19^. **(b-c)** TCNs identified by supervised **(b)** and unsupervised methods **(c)**. **(d)** Left, cells are colored based on 11 cell types. Right, TCNs identified by CytoCommunity. **(e)** Robustness score is defined as the average Jaccard index between the two sets of TCNs generated using the two sets of cell type annotations.

We evaluated the robustness of CytoCommunity with regard to three parameters (Methods): (1) the value of *k* in the *k*-nearest neighbor (*k*-NN)-based cellular spatial graph (Extended Data Fig. 1); (2) the number of GNN models (runs) in the ensemble procedure (Extended Data Fig. 2); and (3) the granularity of cell-type annotation (Extended Data Figs. 3–5). We defined a robustness score as the average Jaccard index^15^ between TCN partitions generated using different parameter values. All robustness assessments were conducted using a mouse hypothalamic preoptic region dataset without sample labels for unsupervised learning and a human triple-negative breast cancer dataset with two classes of samples for supervised learning (Supplementary Table 1).

### Performance evaluation using spatial proteomics data

**Fig. 2:**
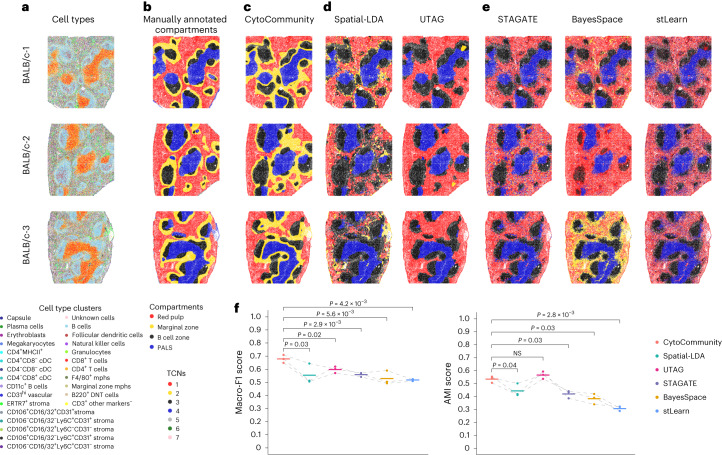
Performance evaluation of the unsupervised CytoCommunity algorithm using single-cell spatial proteomics data. **a**,**b**, Three single-cell spatial images, BALB/c-1, BALB/c-2 and BALB/c-3, generated from healthy mouse spleen samples using the CODEX technology. Cells are colored based on cell-type annotation (**a**) or manual tissue compartment annotation (**b**) from the original study^16^. **c**–**e**, TCNs identified by CytoCommunity (**c**), two methods (Spatial-LDA and UTAG) originally designed for spatial proteomics data (**d**) and three methods (STAGATE, BayesSpace and stLearn) originally designed for spatial transcriptomics data (**e**). **f**, Macro-F1 and AMI scores computed based on manually annotated TCNs. Each data point represents the performance on one image; the horizontal bars represent the mean across *n* = 3 images. Performances (points) on the same image are connected by gray dashed lines. *P* values were computed using a one-sided paired *t*-test. Note that only UTAG identified seven TCNs in the BALB/c-3 image, while all other methods identified four TCNs in all three images. mphs, macrophages; DNT, TCRα^+^CD4^−^CD8^−^ double-negative T [cell]; NS, not significant.

To evaluate the performance of unsupervised CytoCommunity, we applied it to a spatial proteomics dataset of mouse spleen generated using the Co-Detection by Indexing (CODEX) technology^16^ (Supplementary Table 1) and compared it with five state-of-the-art unsupervised learning tools, including Spatial-LDA^9^ and UTAG^10^, which were originally designed for multiplexed imaging data, and STAGATE^8^, BayesSpace^5^ and stLearn^6^, which were originally designed for spatial transcriptomics data (Methods and Supplementary Table 2). The CODEX dataset consists of three healthy mouse spleen samples stained with 30 protein markers (named as BALB/c-1, BALB/c-2 and BALB/c-3). On average, each image contained 81,760 cells covering 27 cell types (Fig. 2a). The images were manually annotated by the authors^16^ into four known tissue compartments of the spleen: red pulp; marginal zone; B cell zone; and the periarteriolar lymphoid sheath (PALS) (Fig. 2b). We regarded these tissue compartments as ground-truth (GT) TCNs. We evaluated the agreement between predicted and GT TCNs using two performance metrics: macro-F1 score and adjusted mutual information (AMI) (Methods). Overall, all six methods can identify the PALS compartment accurately (Fig. 2c–e). However, only CytoCommunity consistently identified the marginal zones (Fig. 2c). Spatial-LDA identified partial and discontinuous marginal zone-like TCNs around B cell zones (Fig. 2d). UTAG identified high-quality red pulp regions but failed to capture any marginal zones (Fig. 2d). The other three methods identified low-quality red pulp regions that intermixed with other types of TCNs (Fig. 2e). Quantitatively, CytoCommunity also achieved the highest macro-F1 score and tied-top AMI score (paired *t*-test, *P* < 0.05) across the three samples (Fig. 2f). In conclusion, CytoCommunity had significantly improved performance over representative state-of-the-art methods when comparing identified TCNs with manually annotated tissue compartments.

### Performance evaluation using spatial transcriptomics data

**Fig. 3:**
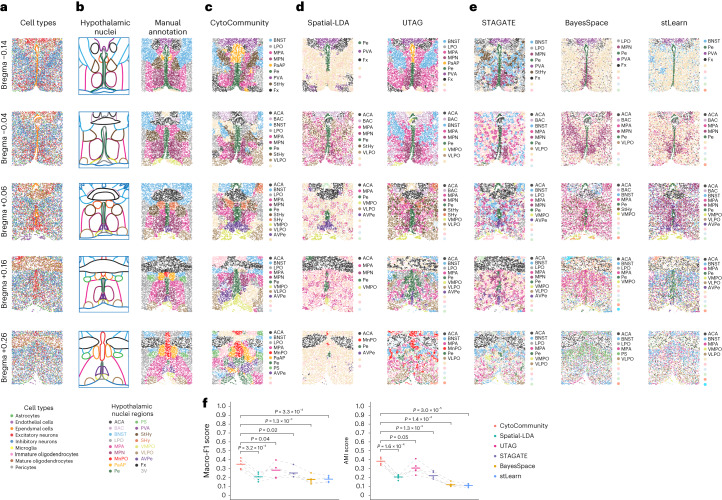
Performance evaluation of the unsupervised CytoCommunity algorithm using single-cell spatial transcriptomics data. **a**, Five single-cell spatial images—Bregma −0.14, Bregma −0.04, Bregma +0.06, Bregma +0.16 and Bregma +0.26—of mouse hypothalamic preoptic region generated using the MERFISH technology. The Bregma distance is given for each imaged brain section. Cells are colored based on the cell-type annotation from the original study^17^. **b**, Left, the 9, 10, 12, 12 and 11 hypothalamic nuclei or regions in the images were manually outlined by the authors of the original study. Right, cells were manually assigned TCN membership based on the nuclei outlined on the left. **c**–**e**, TCNs identified by CytoCommunity (**c**), Spatial-LDA and UTAG (**d**), and STAGATE, BayesSpace and stLearn (**e**). TCNs are labeled and colored based on the most similar manually annotated nuclei regions. TCNs without labels could not be matched to the manual annotation. **f**, Macro-F1 and AMI scores were computed using the manually annotated hypothalamic nuclei in **b**. Each point represents the performance on a given single-cell spatial image; the horizontal bars represent the mean across *n* = 5 images. Performances (points) on the same images are connected by gray dashed lines. *P* values were computed using a one-sided paired *t*-test. 3V, third ventricle; BAC, bed nucleus of the anterior commissure; Fx, fornix; LPO, lateral preoptic area; PS, parastrial nucleus; PVA, paraventricular thalamic nucleus; StHy, striohypothalamic nucleus.

The evaluation above focused on identifying large tissue compartments. To further evaluate the performance of unsupervised CytoCommunity on detecting smaller TCNs, we applied it to two spatial transcriptomic datasets (Supplementary Table 1 and Supplementary Notes). The dataset with more complex tissue structures was generated from the healthy mouse hypothalamic preoptic region using the multiplexed error-robust fluorescence in situ hybridization (MERFISH) technology^17^ to measure the expression of 155 genes. This dataset includes samples from five brain regions: Bregma −0.14; Bregma −0.04; Bregma +0.06; Bregma +0.16; and Bregma +0.26. On average, each image contained 5,352 cells that were assigned to nine cell types by the authors^17^ (Fig. 3a). These images cover 17 hypothalamic nuclei regions manually outlined in the original study based on manual inspection of extensively studied histology of the brain^17,18^ (Fig. 3b, left column). In neuroanatomy, a nucleus is a group of neurons having similar connections and functions. Hence, we treated these manually outlined nuclei as gold standard TCNs in performance evaluation. For quantitative comparison, we generated the GT nuclei annotations for all cells (Fig. 3b, right column) by manually overlaying the outlines of the hypothalamic nuclei onto the single-cell spatial maps. As shown in Fig. 3b, a prominent tissue architectural feature of the preoptic region is the symmetry of several types of nuclei. CytoCommunity identified multiple symmetric and coherent TCNs that agreed with the manually outlined nuclei (Fig. 3c). For example, the symmetric bed nucleus of the stria terminalis (BNST), medial preoptic area (MPA) and medial preoptic nucleus (MPN) regions were identified in all five tissue samples. We also identified symmetric ventrolateral preoptic nucleus (VLPO) regions in Bregma −0.04, Bregma +0.06 and Bregma +0.16, symmetric septohypothalamic nucleus (SHy), anteroventral periventricular nucleus (AVPe) and ventromedial preoptic nucleus (VMPO) regions in Bregma +0.06 and symmetric paraventricular hypothalamic nucleus (PaAP) regions in Bregma +0.26. Besides these symmetric domains, the central anterior commissure (ACA), periventricular hypothalamic nucleus (Pe) and median preoptic nucleus (MnPO) domains were also identified. In comparison, UTAG performed better than CytoCommunity on one sample (Bregma −0.14) with more accurate identification of symmetric and coherent BNST, MPA and MPN (Fig. 3d, right column). Although UTAG performed less well than CytoCommunity on the rest of the samples, it still identified clearer symmetric TCNs than Spatial-LDA (Fig. 3d, left column) and the other three methods originally designed for spatial transcriptomics data (Fig. 3e). These four methods can only identify central ACA and Pe in most samples, but several other nuclei remain unidentified (unlabeled TCNs in the figure legend) because many detected TCNs are intermixed without clear boundary between them and lack clear symmetry (Fig. 3d,e). Quantitatively, CytoCommunity had significantly higher macro-F1 and AMI scores than the other five methods (paired *t*-test *P* ≤ 0.05; Fig. 3f).

Taken together, using the spatial maps generated with different technologies, we demonstrated that CytoCommunity had a significantly improved performance over state-of-the-art methods in identifying TCNs of variable sizes from different tissues.

### Performance evaluation using stratified spatial omics data

**Fig. 4:**
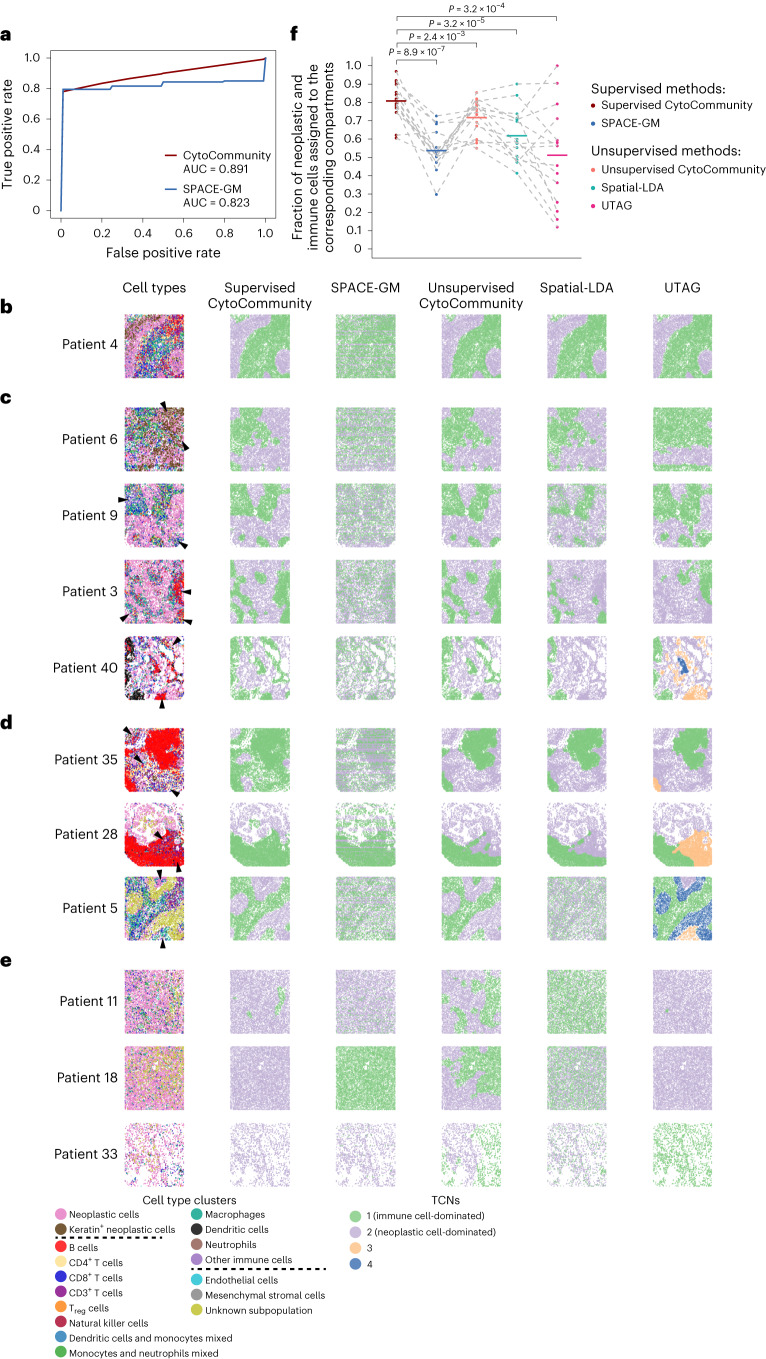
Performance evaluation of the supervised CytoCommunity algorithm using stratified single-cell spatial proteomics data. **a**, ROC curves for the image label (compartmentalized versus mixed tumors) prediction. The AUCs for CytoCommunity and SPACE-GM represent the mean values of ten sets of tenfold cross-validations. **b**–**e**, Representative single-cell images of the compartmentalized (**b**–**d**) and mixed (**e**) tumors from patients with triple-negative breast cancer. Cells are colored based on the cell-type annotation from the original study^19^ (first column) or TCNs identified using two supervised methods, supervised CytoCommunity and SPACE-GM, and three unsupervised methods, that is, unsupervised CytoCommunity, Spatial-LDA and UTAG. **b**, Tissue image of patient no. 4 on which all methods showed good performance, except for SPACE-GM. **c**, Tissue images on which supervised and unsupervised CytoCommunity showed better performance than three other methods. **d**, Tissue images on which supervised CytoCommunity showed better performance than all other methods. Mis-assigned regions by the compared methods are indicated by the arrowheads in the cell-type maps. **e**, Representative single-cell images of the mixed tumors from patients with triple-negative breast cancer. **f**, Fractions of neoplastic and immune cells correctly assigned to the neoplastic cell-dominated and immune cell-dominated TCNs. Each point represents performance on a given compartmentalized tumor image; the horizontal bars represent the mean across *n* = 15 images. Performances (points) on the same images are connected by gray dashed lines. *P* values were computed using a one-sided paired *t*-test. Note that the number of TCNs were set to two for CytoCommunity, SPACE-GM and Spatial-LDA. Clustering resolution was set to 0.05 for UTAG, resulting in one or two identified TCNs in most images but three or four identified TCNs in the rest of the images.

To demonstrate the advantage of supervised CytoCommunity to identify condition-specific TCNs, we applied it to a stratified spatial proteomics dataset of 41 patients with triple-negative breast cancer generated using the multiplexed ion beam imaging by time-of-flight (MIBI-TOF) technology^19^ (Supplementary Table 1). This dataset consisted of 15, 19 and 6 MIBI-TOF images from compartmentalized (characterized by immune cells spatially segregated from neoplastic cells), mixed (characterized by a high degree of intermixing of neoplastic and immune cells) and cold (characterized by a low degree of immune cell infiltration) tumors, respectively^19^. We asked whether supervised CytoCommunity could identify spatially separated neoplastic and immune cell-dominated regions in the compartmentalized tumors. To this end, we used MIBI-TOF images from compartmentalized and mixed tumors as the input to supervised CytoCommunity and compared its performance with SPACE-GM, unsupervised CytoCommunity, Spatial-LDA and UTAG. We chose Spatial-LDA and UTAG because these two methods worked well for single-cell spatial proteomics data. The images from cold tumors were not used because of a small number of immune cells in the tumors. We first evaluated the performance of the two supervised methods to predict tumor phenotypes (that is, compartmentalized versus mixed tumors). Using ten sets of tenfold cross-validation (Methods), CytoCommunity showed improved prediction performance, with an average area under the receiver operating characteristic curve (AUCROC) of 0.891, compared to an average AUC of 0.823 using SPACE-GM (Fig. 4a).

Next, we evaluated the TCN detection performance of all methods using the fraction of cells assigned to the correct compartments as the performance metric (that is, the assignment of immune cells to immune cell-dominated TCNs and vice versa). Unexpectedly, SPACE-GM performed poorly with no coherent TCNs identified in the compartmentalized tumors (Fig. 4b–d, third column and Extended Data Fig. 4b), suggesting that TCNs identified by SPACE-GM do not represent coherent tissue structures. Instead, they may represent ‘spatial motifs’ of smaller sizes as defined in the original study^11^. For the other four methods, although all of them correctly identified neoplastic cell-dominated or immune cell-dominated TCNs that are consistent with compartmentalized architecture in a few patient samples (for example, patient 4; Fig. 4b), for the majority of patient samples, supervised CytoCommunity showed superior performance over the three unsupervised methods (Fig. 4c,d and Extended Data Fig. 4b,c). For example, for patient 6, supervised and unsupervised CytoCommunity and Spatial-LDA identified similar TCNs that are consistent with the spatial distribution patterns of cell types. However, UTAG mis-assigned many neoplastic cells to the immune cell-dominated TCN (Fig. 4c, arrowheads, first row). For patient 9, supervised and unsupervised CytoCommunity and UTAG identified clearly separated immune cell-dominated and neoplastic cell-dominated TCNs, but Spatial-LDA identified two intermixed TCNs without clear boundaries (Fig. 4c, arrowheads, second row). For patient 3 and 40, supervised and unsupervised CytoCommunity identified the correct TCNs, but both Spatial-LDA and UTAG mis-assigned many immune cells to neoplastic cell-dominated TCNs (Fig. 4c, arrowheads, third and fourth rows).

All three unsupervised methods performed poorly on samples having complex cell-type compositions at the neoplastic–immune boundaries (Extended Data Fig. 4c). For instance, they all mis-assigned a small band and a relatively large immune cell-enriched regions, respectively, located at the top left and bottom right of the image of patient 35 (Fig. 4d, arrowheads, top row) to neoplastic cell-dominated TCNs. Similarly, both unsupervised CytoCommunity and Spatial-LDA assigned a region at the bottom right of the tissue containing B cells, CD3^+^ T cells, CD4^+^ T cells, CD8^+^ T cells and neutrophils (Fig. 4d, arrowheads, middle row) to neoplastic cell-dominated TCNs in patient 28, while UTAG identified this region as a third TCN. In contrast, supervised CytoCommunity successfully distinguished neoplastic cell-dominated and immune cell-dominated TCNs in these two patients. For patient 5, there were fewer neoplastic cells and a region enriched for an unknown subpopulation with clear boundaries in the tissue (Fig. 4d, arrowhead, bottom row). Given this more complicated tissue architecture, unsupervised CytoCommunity assigned neoplastic cells at the top of the tissue (Fig. 4d, top arrowhead, bottom row) and immune cells to one TCN and the unknown subpopulation as another TCN, while UTAG identified four TCNs, separating immune cells into two TCNs. Spatial-LDA predicted two intermixed TCNs that were inconsistent with manual inspection. In contrast, only supervised CytoCommunity correctly identified the immune cell-dominated TCN with the fewest neoplastic cells included, although it assigned the unknown subpopulation to the neoplastic cell-dominated TCN.

Regarding mixed tumors, supervised CytoCommunity and SPACE-GM often identified two TCNs with very different sizes or just a single TCN (Fig. 4e and Extended Data Fig. 5b), which is consistent with the architecture of highly intermixed neoplastic and immune cells. In comparison, unsupervised CytoCommunity and Spatial-LDA cannot use sample label information and were forced to identify two TCNs in all mixed tumors, which are similar to TCNs identified in compartmentalized tumors (Fig. 4e and Extended Data Fig. 5c).

To quantify the performance, we computed the fraction of neoplastic and immune cells that were correctly assigned to neoplastic cell-dominated and immune cell-dominated TCNs on compartmentalized tumor samples. Supervised CytoCommunity had a significantly better performance than all compared supervised and unsupervised methods (all paired *t*-test *P* < 0.005; Fig. 4f).

### Risk-specific immune-associated TCNs in colorectal cancer

**Fig. 5:**
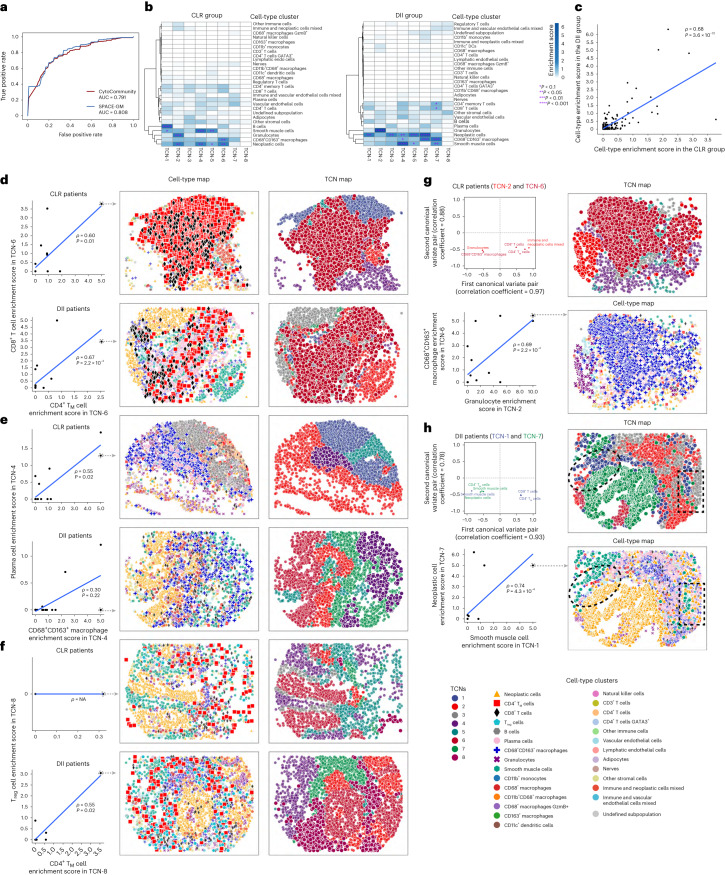
Coordinated neoplastic and immune cell-type distributions within or between TCNs in CRC. **a**, ROC curves for image label (CLR versus DII) prediction. The AUCs for CytoCommunity and SPACE-GM represent the mean values of ten sets of tenfold cross-validations. **b**, Heatmaps of the average enrichment scores of each cell type in each identified TCN across all images of the CLR and DII patient samples. Cell type enrichment score was defined as −log_10_(*P*). *P* values were computed using hypergeometric tests and adjusted with the Benjamini–Hochberg method^45^. **c**, Correlation of average cell-type enrichment scores in all identified TCNs between CLR and DII patients. **d**–**f**, Correlation of the enrichment scores of two indicated cell types in TCN-6 (**d**), TCN-4 (**e**) or TCN-8 (**f**) in each patient group (left). Representative cell-type and TCN maps (middle and right) are based on patient samples indicated by a dashed circle in the scatter plots. **g**,**h**, Significant canonical correlation (permutation test *P* < 0.1) between two TCNs in the CLR (**g**) and DII (**h**) patient groups. Scatter plots of normalized weights of dominant cell types (observed variable) in each TCN in the first two canonical variate pairs (top left) are shown. Correlation of the enrichment scores of dominant cell types in the first canonical variate pair (bottom left), and representative cell-type and TCN maps (right), are also shown. The black dashed ellipses and rectangles in the cell-type and TCN maps in **h** are used to highlight the colocalization of smooth muscle cells in TCN-1 and neoplastic cells in TCN-7. For all scatter plots, regression lines, Spearman rank correlation coefficients (*ρ*) and two-sided Spearman *ρ* test *P* values are shown. For clarity, cells of the studied types and TCNs have been magnified without transparency in all cell-type and TCN maps.

To demonstrate the utility of supervised CytoCommunity for de novo identification of condition-specific TCNs using supervised learning, we applied it to a CODEX dataset generated using samples from 17 low-risk (Crohn’s-like lymphoid reaction (CLR)) and 18 high-risk (diffuse inflammatory infiltration (DII)) patients with colorectal cancer (CRC)^3^ (Supplementary Table 1). The CLR patient group had significantly better overall survival (OS) than the DII patient group (log-rank test *P* = 0.002)^3^. The dataset consisted of 68 and 72 CODEX images from the CLR and DII patients, respectively. Using ten sets of tenfold cross-validation, we found that CytoCommunity classified the images into the two patient groups with an average AUC of 0.791, compared to SPACE-GM, with an average AUC of 0.808 (Fig. 5a).

**Extended Data Fig. 6:**
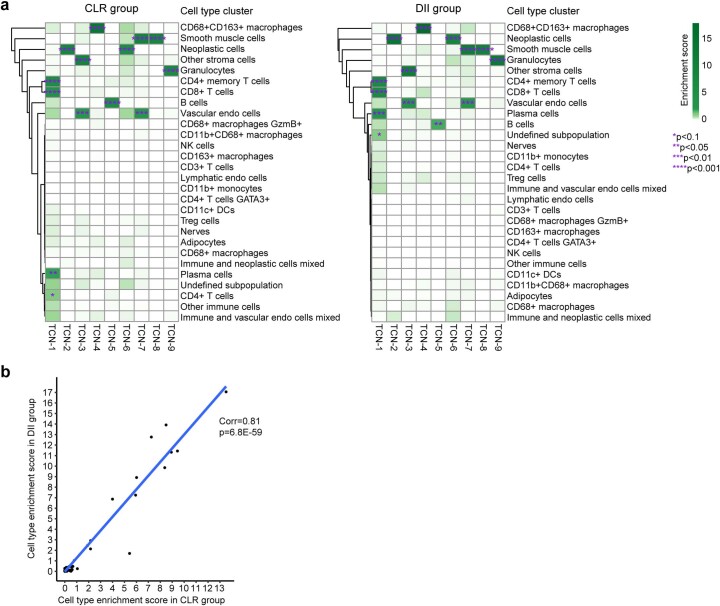
Analysis of TCNs in colorectal cancer identified by the original study^3^. **(a)** Heatmaps of average enrichment scores of each cell type in each identified TCN across all tissue sections of CLR and DII patient samples. Cell type enrichment score is defined as -log_10_(*P*-value). *P*-values were computed using hypergeometric tests and adjusted using Benjamini-Hochberg method. **(b)** Spearman’s rank correlation coefficient (Corr) of average cell type enrichment scores in all identified TCNs between CLR and DII patients and the two-sided Spearman’s rho test *p*-value are shown.

**Extended Data Fig. 7:**
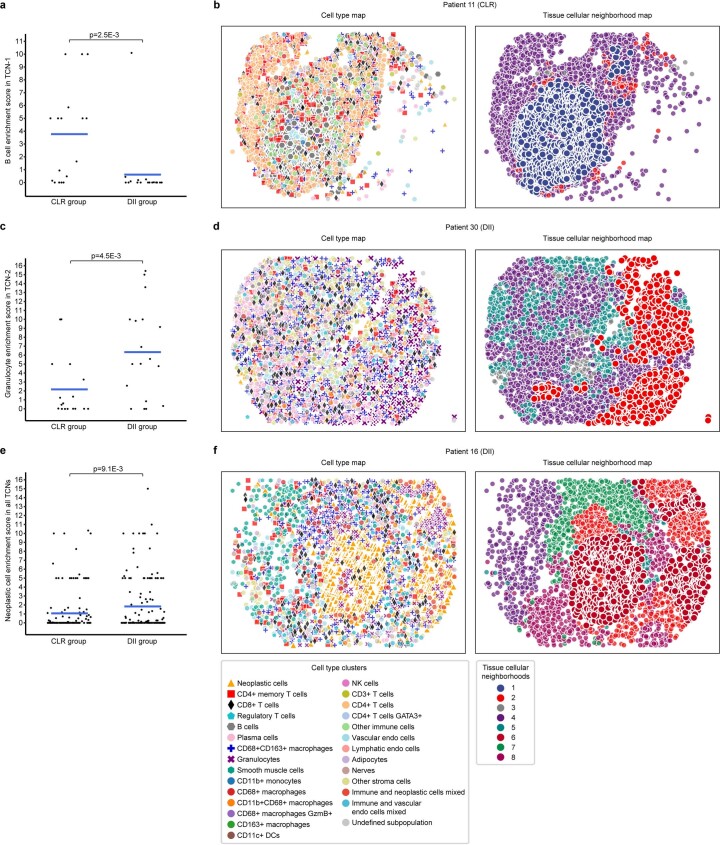
Representative TCNs specific to low- and high-risk colorectal cancer patients. **(a)** Comparison of B cell enrichment scores in TCN-1 between CLR patients and DII patients (n = 17 and 18 for the CLR and DII groups, respectively). **(b)** Cell type and TCN maps of a representative CLR patient image with B cells enriched in TCN-1. **(c)** Comparison of granulocyte enrichment scores in TCN-2 between CLR patients and DII patients (n = 17 and 18 for the CLR and DII groups, respectively). **(d)** Cell type and TCN maps of a representative DII patient image with granulocytes enriched in TCN-2. **(e)** Comparison of neoplastic cell enrichment scores in all TCNs between CLR patients and DII patients (n = 136 and 144 for the CLR and DII groups, respectively). **(f)** Cell type and TCN maps of a representative DII patient image with neoplastic cells enriched in TCN-6. In all jitter plots, horizontal bars represent the means across data points. *P*-values were computed using one-sided *t*-tests. For clarity, cells of studied types and TCNs are shown in larger size without transparency in all cell type and TCN maps.

We next investigated the eight TCNs identified using supervised CytoCommunity and found that the cell-type enrichment scores (Methods) in those TCNs were significantly correlated (Spearman rank *ρ* = 0.68; Fig. 5b,c) between the two patient groups. We also analyzed the cell-type enrichment of TCNs reported in the original study^3^. The correlation (0.81) of enriched cell types between the two patient groups was substantially higher than our TCNs (Extended Data Fig. 6), suggesting that the original study missed some condition-specific TCNs. In comparison, we found several cell types that were enriched in CLR-specific or DII-specific TCNs identified by CytoCommunity. For example, B cells were significantly enriched in TCN-1 in CLR patients but not in any TCN in DII patients (*t*-test *P* = 2.5 × 10^−3^; Fig. 5b and Extended Data Fig. 7a,b), which is consistent with the presence of B cell-enriched tertiary lymphoid structures in the CLR patient samples but not in the DII patient samples^3^. On the other hand, granulocytes were more significantly enriched in TCN-2 in DII patients than in CLR patients (*t*-test *P* = 4.5 × 10^−3^; Fig. 5b and Extended Data Fig. 7c,d), which is consistent with previous reports that neutrophils have a tumor-promoting role^20,21^. Interestingly, neoplastic cells were more significantly enriched in TCNs in the DII group than in the CLR group (*t*-test *P* = 9.1 × 10^−3^; Fig. 5b and Extended Data Fig. 7e,f), suggesting a more active role played by neoplastic cells in shaping the tumor microenvironment (TME) in high-risk patients with cancer. These condition-specific TCNs were not reported in the original study (Extended Data Fig. 6a).

Besides enrichment of individual cell types in TCNs, we also investigated the coordination of cell types within and between TCNs to better understand cell–cell communication in the TME. As an example of within-TCN cell-type communication shared by the two patient groups (Supplementary Fig. 1), the enrichment of CD4^+^ memory T (T_M_) cells (red square) was significantly correlated with CD8^+^ T cell (black diamond) enrichment in TCN-6 in both CLR (*ρ* = 0.60) and DII (*ρ* = 0.67) patients (Fig. 5d, left). Consistent with the cell-type and TCN maps from patients with high enrichment scores, we observed that the two cell types were intermixed with each other in TCN-6 (Fig. 5d, middle and right). We also found CLR-specific (Supplementary Fig. 1a) and DII-specific (Supplementary Fig. 1b) cell-type associations within TCNs. For instance, the enrichment of CD68^+^CD163^+^ macrophages (blue plus sign) was significantly correlated with plasma cell (pink octagon) enrichment in TCN-4 in CLR patients (*ρ* = 0.55) but not in DII patients (Fig. 5e), suggesting that double-positive macrophages have an antitumor effect by promoting plasma cell enrichment to improve the survival of CLR patients. Previous studies demonstrated that human macrophages can induce the differentiation of B cells into plasma cells^22^, which may secrete antibodies that promote antitumor immune responses^23^. As an opposite example, the enrichment of CD4^+^ T_M_ (red square) and regulatory T (T_reg_) cells (cyan pentagon) in TCN-8 had a significant correlation (*ρ* = 0.55) in DII patients but not in CLR patients (Fig. 5f). This is in line with previous reports that CD4^+^ T cells can be transformed into T_reg_ cells^24^, resulting in an immunosuppressive TME and poor patient survival.

To investigate the communication between different TCNs, we conducted canonical correlation analysis of TCN pairs (Methods). We found substantial differences in significant canonical correlations of TCNs between CLR (Supplementary Fig. 2a) and DII patients (Supplementary Fig. 2b). As an example of significant between-TCN associations specific to CLR patients, the granulocytes and immune and neoplastic cell mixed subpopulation in TCN-2, and CD68^+^CD163^+^ macrophages, CD4^+^ T_M_ and CD8^+^ T cells in TCN-6 were the dominant cell types (observed variables) in the first canonical variate pair (Fig. 5g, top left). Without consideration of other cell types, granulocytes (purple cross) and CD68^+^CD163^+^ macrophages (blue plus sign) in the two TCNs had a significant correlation (*ρ* = 0.69; Fig. 5g, bottom left), suggesting a potential interaction between these two cell types across TCNs. Consistent with the corresponding cell-type and TCN maps, we observed that granulocytes enriched in TCN-2 were close to double-positive macrophages enriched in TCN-6 (Fig. 5g, right). Such between-TCN communication in CLR patients is supported by previously observed interactions between neutrophils and macrophages that could exert an antitumor effect^25^.

Another interesting example of between-TCN communication regarding the DII group is the significant association between TCN-1 and TCN-7, in which smooth muscle cells, CD4^+^ T_M_ cells and CD8^+^ T cells in TCN-1, and neoplastic, CD4^+^ T_M_ cells and smooth muscle cells in TCN-7 were the dominant cell types in the first canonical variate pair (Fig. 5h, top left). By examining the pair-wise correlation of these cell types, we found that smooth muscle cells (green hexagon) in TCN-1 were significantly correlated with malignant cells (orange triangle) in TCN-7 (*ρ* = 0.74; Fig. 5h, bottom left). As supporting evidence, previous studies reported the critical role of smooth muscle cells in intestinal architecture and vascular function^26^, tumor angiogenesis and metastasis^27^, which is consistent with our observation that malignant cells in TCN-7 are spatially close to smooth muscle cells in TCN-1 (dashed ellipses and rectangles, Fig. 5h, right).

### Risk-specific stromal-associated TCNs in breast cancer

**Fig. 6:**
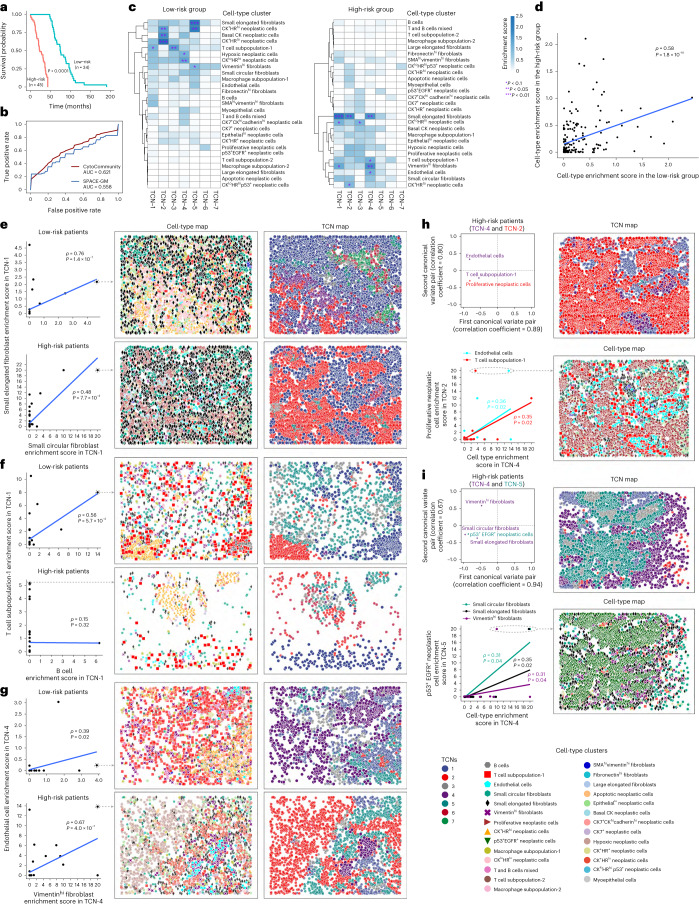
Coordinated neoplastic and stromal cell-type distributions within or between TCNs in breast cancer. **a**, Kaplan–Meier survival curves of 79 patients with breast cancer who were classified into low-risk and high-risk groups based on their median OS time. The *P* value was computed using the log-rank test. **b**, ROC curves for the image label (low-risk versus high-risk) prediction. The AUC values for CytoCommunity and SPACE-GM represent the mean values of ten sets of tenfold cross-validations. **c**, Heatmaps of the average enrichment scores of each cell type in each identified TCN across all images of low-risk and high-risk patient samples. Cell type enrichment score was defined as −log_10_(*P*). *P* values were computed using hypergeometric tests and adjusted using the Benjamini–Hochberg method^45^. **d**, Correlation of average cell-type enrichment scores in all identified TCNs between low-risk and high-risk patients. **e**–**g**, Correlation of the enrichment scores of two indicated cell types in TCN-1 (**e** and **f**) or TCN-4 (**g**) in each patient group (left). Representative cell-type and TCN maps (middle and right) are based on patient samples indicated by a dashed circle in the scatter plots. **h**,**i**, Significant canonical correlation (permutation test *P* < 0.1) between TCN-4 and TCN-2 (**h**) and between TCN-4 and TCN-5 (**i**) in the h**i**gh-risk patient group. The scatter plots of normalized weights of the dominant cell types (observed variable) in each TCN in the first two canonical variate pairs (top left) are shown. Correlation of the enrichment scores of the dominant cell types in the first canonical variate pair (bottom left), and representative cell-type and TCN maps (right), are also shown. For all scatter plots, regression lines, Spearman rank correlation coefficients (*ρ*) and two-sided Spearman *ρ* test *P* values are shown. For clarity, cells of the studied types and TCNs have been magnified without transparency in all cell-type and TCN maps.

To further evaluate the ability of supervised CytoCommunity to discover condition-specific TCNs using different data modalities, we applied it to another spatial proteomics dataset of breast cancer generated using the imaging mass cytometry (IMC) technology^12^ (Supplementary Table 1). Based on the median OS, we stratified 79 breast cancer patients into low-risk and high-risk groups with significant survival difference (log-rank test *P* < 0.0001; Fig. 6a and Methods). Using these patient labels, we evaluated the performance of supervised CytoCommunity and SPACE-GM to classify the images into two patient prognosis groups. We found that CytoCommunity achieved improved prediction performance with an average AUC of 0.621, compared to an average AUC of 0.558 by SPACE-GM (Fig. 6b).

**Extended Data Fig. 8:**
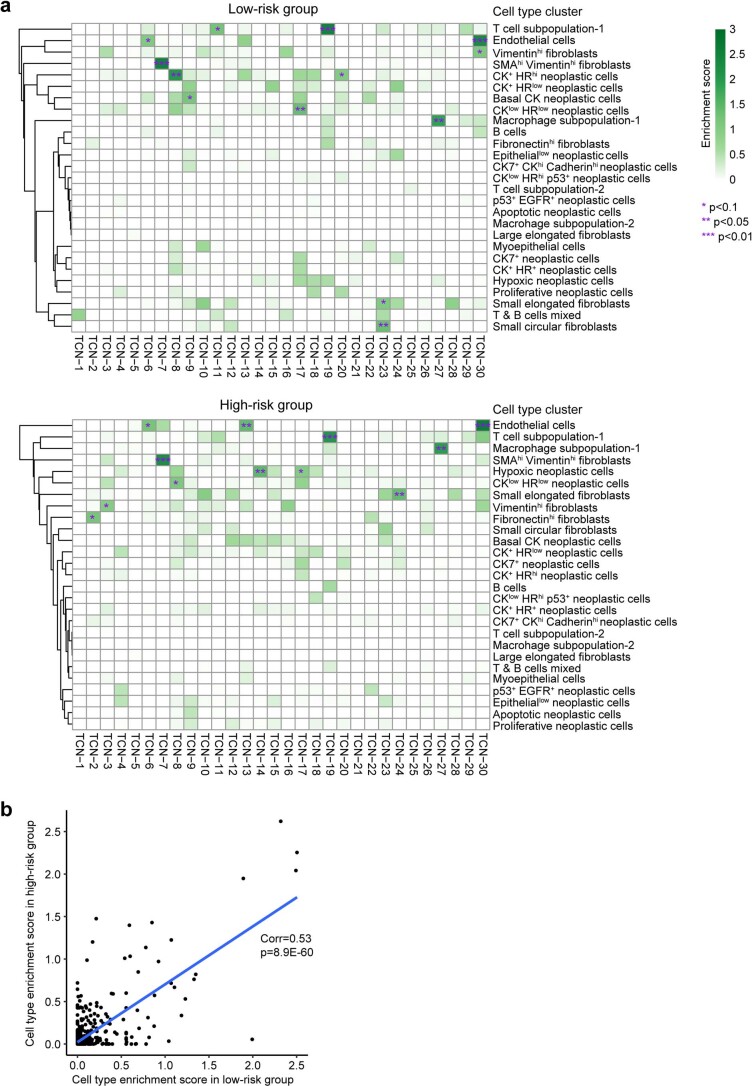
Analysis of TCNs in breast cancer identified by the original study^12^. **(a)** Heatmaps of average enrichment scores of each cell type in each identified TCN across all tissue sections of low-risk and high-risk patient samples. Cell type enrichment score is defined as -log_10_(*P*-value). *P*-values were computed using hypergeometric tests and adjusted using Benjamini-Hochberg method. **(b)** Spearman’s rank correlation coefficient (Corr) of average cell type enrichment scores in all identified TCNs between low-risk and high-risk patients and the two-sided Spearman’s rho test *p*-value are shown.

**Extended Data Fig. 9:**
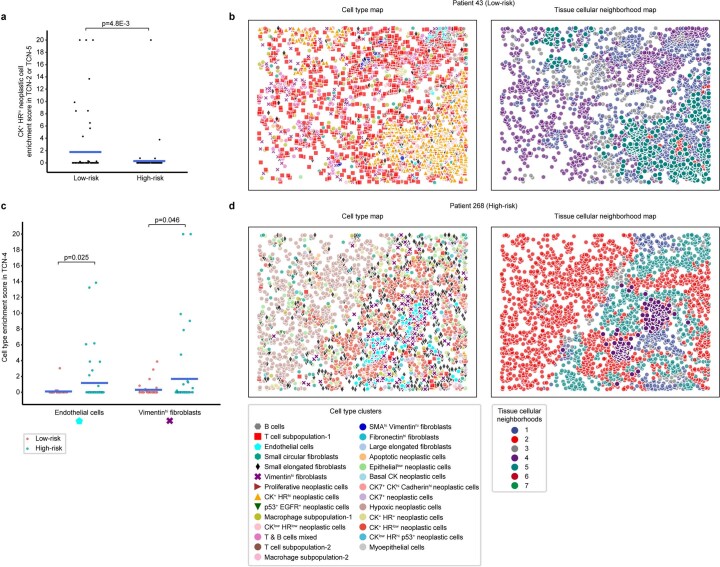
Representative TCNs specific to low- and high-risk breast cancer patients. **(a)** Comparison of CK^+^ HR^hi^ neoplastic cell enrichment scores in TCN-2 or TCN-5 between low-risk and high-risk patients (n = 68 and 90 for the low- and high-risk groups, respectively). **(b)** Cell type and TCN maps of a representative low-risk patient with CK^+^ HR^hi^ neoplastic cells enriched in TCN-5. **(c)** Comparison of endothelial cell and Vimentin^hi^ fibroblast enrichment scores in TCN-4 between low-risk and high-risk patients (n = 34 and 45 for the low- and high-risk groups, respectively). **(d)** Cell type and TCN maps of a representative high-risk patient with both endothelial cell and Vimentin^hi^ fibroblast enriched in TCN-4. In all jitter plots, horizontal bars represent the means across data points. *P*-values were computed using one-sided *t*-tests. For clarity, discussed cell types and TCNs are shown in larger size without transparency in all cell type and TCN maps.

**Extended Data Fig. 10:**
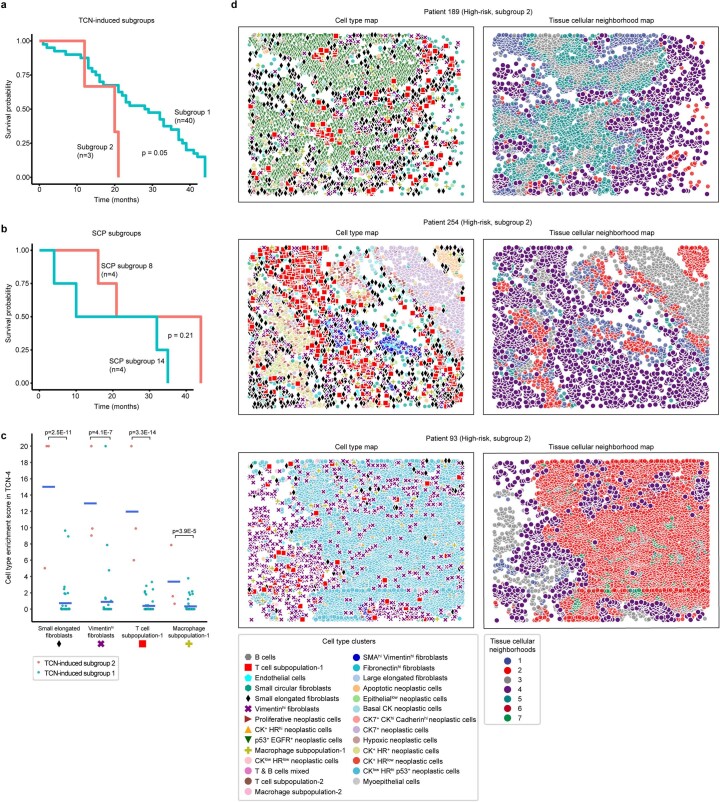
Prognosis ability of TCNs identified in breast cancer. **(a)** Kaplan-Meier survival curves of high-risk breast cancer patients who were clustered into three subgroups based on cell type enrichment scores of TCNs. Subgroup 3 was removed from the survival analysis since it does not contain more than three patients. **(b)** Kaplan-Meier survival curves of high-risk breast cancer patients who were assigned into 18 single-cell pathology (SCP) subgroups by the original study^12^. Two SCP subgroups with more than three patients were used for survival analysis. *P*-values between survival curves were computed using the log-rank test. **(c)** Comparison of four cell type (including small elongated fibroblasts, Vimentin^hi^ fibroblasts, T cells and macrophages) enrichment scores in TCN-4 between high-risk patients (points) in the TCN-induced subgroup 1 and subgroup 2 (shown in panel a). Horizontal bars represent the means across patients. *P*-value was computed using one-sided *t*-test (n = 40 and 3 for the TCN-induced subgroup 1 and subgroup 2, respectively). **(d)** Cell type and TCN maps of three patients in TCN-induced subgroup 2 with the four cell types (shown in panel c) significantly enriched in TCN-4. For clarity, cells of studied types and TCNs are shown in larger size without transparency in all cell type and TCN maps.

We further analyzed the seven TCNs in both the low-risk and high-risk groups identified by CytoCommunity. By comparing the cell-type enrichment scores (Fig. 6c,d), we found that TCNs in both groups have similar overall cell-type composition (*ρ* = 0.58; Fig. 6d). We also analyzed cell-type enrichment of TCNs reported in the original study and found a similar moderate correlation of cell-type enrichment scores between the low-risk and high-risk patient groups (*ρ* = 0.53; Extended Data Fig. 8). The TCNs identified by CytoCommunity were enriched for several types of fibroblasts (Fig. 6c), suggesting a critical role of fibroblasts in breast cancer prognosis. Specifically, we found that both vimentin^hi^ cancer-associated fibroblasts (CAFs) and endothelial cells were more enriched in TCN-4 of the high-risk group than those of the low-risk group (both *t*-test *P* < 0.05; Extended Data Fig. 9c,d). This condition-specific TCN was not identified by the original study (Extended Data Fig. 8a). Besides stromal cell types, we also found low-risk group-specific TCNs (TCN-2 and TCN-5) characterized by the enrichment of CK^+^HR^hi^ neoplastic cells (*t*-test *P* = 4.8 × 10^−3^; Fig. 6c and Extended Data Fig. 9a,b). This is consistent with the previous report that this malignant cell phenotype is associated with good prognosis^12^; such a condition-specific TCN was also captured by the original study (Extended Data Fig. 8a). We compared the prognosis power of TCNs identified by CytoCommunity and the original study (Methods) and found a small subgroup of high-risk patients (subgroup 2) with poorer outcome compared to most high-risk patients (subgroup 1; Extended Data Fig. 10a). We further found significant differences between the two subgroups with respect to small elongated fibroblast, CAF, T cell and macrophage enrichment in TCN-4 (Extended Data Fig. 10c,d), suggesting a non-negligible impact of normal fibroblasts and CAFs on immune cells in patients with breast cancer, resulting in an unfavorable prognosis. No significant survival difference among the high-risk patients was captured by single-cell pathology-based subtyping^12^ (Extended Data Fig. 10b).

Regarding cell-type association within TCNs (Supplementary Fig. 3), we found that two normal fibroblast types, small circular (green hexagon) and elongated (black diamond) fibroblasts, were significantly correlated in TCN-1 in both low-risk (*ρ* = 0.76) and high-risk (*ρ* = 0.48) patients (Fig. 6e, left). We observed that these two fibroblast types were intermixed in TCN-1 in patients with high enrichment scores (Fig. 6e, middle and right). As examples of low-risk-specific (Supplementary Fig. 3a) and high-risk-specific (Supplementary Fig. 3b) within-TCN cell–cell communication, we found that B cell (gray hexagon) enrichment was significantly correlated with T cell (red square) enrichment in TCN-1 in low-risk patients (*ρ* = 0.56; Fig. 6f), but not in high-risk patients. Previous studies revealed that B cells can induce T cell activation and proliferation to exert antitumor effects^28^. In contrast, we found that CAFs (purple cross) and endothelial cells (cyan pentagon) had a strong correlation in TCN-4 in high-risk patients (*ρ* = 0.67; Fig. 6g), but not in low-risk patients. This is in line with previous reports that CAFs can regulate endothelial cell function and promote angiogenesis^29,30^ to facilitate cancer metastasis, leading to unfavorable patient outcomes.

Next, we investigated between-TCN communication by canonical correlation analysis. We found several significant TCN associations involving neoplastic-stromal cell interactions in both low-risk and high-risk patients (Supplementary Fig. 4). For example, we observed that endothelial cell (cyan pentagon) and T cell (red square)-dominated TCN-4 was associated with TCN-2, which was dominated by proliferative neoplastic cells (brown right-pointing triangle) (Fig. 6h). As supporting evidence, previous studies showed that endothelial cell-mediated neovascularization can not only transport oxygen and nutrients to support rapid proliferation of neoplastic cells^31^, but also serve as a barrier to block T cell infiltration into the tumor bed^32^. Another interesting example is the significant correlation between p53^+^ epidermal growth factor receptor (EGFR)^+^ neoplastic cell (green upside-down triangle)-dominated TCN-5 and TCN-4, which mainly consists of small circular (green hexagon) and elongated fibroblasts (black diamond), as well as CAFs (purple cross) (Fig. 6i). p53^+^EGFR^+^ is a common characteristic of the malignant cells of triple-negative breast cancer^12^, which has a highly aggressive and hypoxic phenotype compared to other breast cancer subtypes^33^. Hypoxia enables the expansion of aggressive tumor clones^34^ (represented by the cohesive tumor mass in TCN-5), and can also support the transformation of tissue-resident fibroblasts into CAFs^34^ in TCN-4, which could in turn promote angiogenesis, as shown in Fig. 6g, or block immune cells from entering the tumor nests as shown in Extended Data Fig. 10d, resulting in poor prognosis in breast cancer.

## Discussion

CytoCommunity identified the TCN as a community detection problem on node-attributed cell–cell spatial proximity graphs. As most traditional community detection algorithms focus only on graph topology to find densely connected subgraphs and cannot explicitly deal with node attributes^35^, CytoCommunity uses a MinCut-based GNN model to learn optimal TCN assignment of cells from cell-type information. Unlike previous methods^2,4–11^, CytoCommunity is the first TCN detection method that can be applied in both unsupervised and supervised modes. This unique feature of CytoCommunity can be attributed to the use of differentiable graph pooling that preserves TCN partition information in the embedding representation of the whole spatial map and thus addresses TCN alignment across spatial maps by training an end-to-end model to classify samples. In contrast, existing unsupervised methods use ad hoc strategies to identify condition-specific tissue domains by aligning TCNs detected on each map according to their spatial positions and then conducting a post hoc comparison across them. It is also worth noting that TCN identification under the supervision of sample labels is a weakly supervised graph partitioning problem, representing an interesting research topic in graph learning.

We believe that the success of CytoCommunity can be attributed to three main features. First, it leverages a GNN model with a theoretically grounded MinCut-based loss function^14^ for soft TCN assignment learning, generating more accurate and stable graph partitioning results than other pooling-capable GNN models, such as DiffPool^36^, which uses heuristic loss functions to learn the soft assignments. Second, CytoCommunity uses a differentiable graph pooling layer to exploit the soft TCN assignment matrix to coarsen the input graph and generate the embedding of the whole graph that is used for sample or image classification. Such framework enables effective learning of condition-specific TCN assignments using the sample labels in an end-to-end fashion. Third, CytoCommunity uses cell types as the initial cell features, probably leading to a better measurement of functional similarity between cells than using noisy gene or protein expression data directly. Cell-type identification is typically the first crucial task in single-cell data analysis and often needs sophisticated tools^37–39^ as well as expert knowledge. Therefore, cell-type annotation should be directly used in a specialized TCN detection method rather than starting with expression data. CytoCommunity encodes cell types in a categorical vector space and thus has scalability to incorporate more heterogenous categorical data, such as cell states^40^, into the initial cell feature vectors to infer TCNs.

Because of the use of cell-type information, the current version of CytoCommunity is not directly applicable to spatial transcriptomics data with spot resolution^41,42^. To address this issue, cell-type composition at each spot can be first estimated using deconvolution^43,44^. Then, a spot–spot proximity graph with inferred cell-type fractions as node attributes can be constructed as the input to CytoCommunity for TCN identification. However, because of the dependence of the CytoCommunity performance on cell-type deconvolution methods, users should test different deconvolution methods to obtain optimal TCN partitions (Supplementary Notes). Another limitation of CytoCommunity is the moderate accuracy of sample class prediction by its supervised version. This can be improved by integration with paired histological images that complement cellular morphology features. Finally, detection of TCN evolution should be considered in the future development of CytoCommunity to capture the dynamics of tissue organization in disease development and progression using spatiotemporal omics data.

In summary, with the rapid growth of single-cell spatial omics maps, CytoCommunity represents a powerful and scalable method for de novo identification of condition-specific TCNs. TCNs directly learned from cell types can facilitate the interpretation of their function and the discovery of cell–cell communication within the tissue microenvironment.

## Methods

### Unsupervised model for the identification of TCNs

The CytoCommunity algorithm consists of two components: a soft TCN assignment learning module and a TCN ensemble module to determine the final robust TCNs (Fig. 1a). In the first component, given a single-cell spatial map with cell-type annotation and cell spatial coordinates, an undirected *k*-NN graph (that is, cellular spatial graph) with node attribute (cell type) is constructed. A cell is represented by a node and its cell-type information is represented by a node attribute vector using one-hot encoding (Fig. 1a, top). We first constructed a directed *k*-NN graph by connecting each node to its *k*-NNs based on Euclidean distance calculated using cell spatial coordinates. Then, the underlying undirected graph without self-edges derived from the directed *k*-NN graph was considered as the cellular spatial graph, which was the input to the GNN model. Because each spatial omics dataset is measured from the same tissue type with the same technology, we set the default value of *k* in the *k*-NN graphs as the square root of the average number of cells (square root of mean (SRM)) across spatial maps in the dataset based on our extensive testing. We also evaluated the effect of different *k* values on the robustness of the algorithm by varying the value of *k* around the SRM (Extended Data Fig. 1) and demonstrated that TCN partitions generated using different *k* values were robust.

As shown in Fig. 1a (top), given a cellular spatial graph with an adjacency matrix $A\in {\{0,1\}}^{n\times n}$ and a node attribute matrix $F\in {\{0,1\}}^{n\times m}$ as the input, we used a basic graph convolution layer^46^ with the rectified linear unit (ReLU) activation function to generate a cell node embedding matrix $X\in {{\mathbb{R}}}^{n\times d}$, where *n* is the number of nodes in the cellular spatial graph and *m* is the number of cell types. Each row of *X* is a learned *d*-dimensional representation vector of a node defined as below:

1 $${{{\mathbf{x}}}}_{i}^{{\prime} }={\rm{ReLU}}({\Theta }_{1}{{{\mathbf{x}}}}_{i}+{\Theta }_{2}\sum _{j\in N(i)}{{{{\mathbf{x}}}}}_{j})$$

where ${{{\mathbf{x}}}}_i^{{{{\prime} }}}$ is an updated embedding vector of cell node *i*, which is calculated based on the previous representation of itself **x**_*i*_ and its first-order neighborhood *N*(*i*) derived from the matrix *A*. **x**_*i*_ is initialized with the node attribute vector (that is, the *i*-th row of the matrix *F*). *Θ*_1_ and *Θ*_2_ are trainable parameter matrices in the graph convolution layer. The value of *d* was empirically set to 128 for the unsupervised tasks and 512 for the supervised tasks in this study.

Next, we use a fully connected neural network with no hidden layer, also known as a linear layer, and the softmax activation function to transform the node embedding matrix $X\in {{\mathbb{R}}}^{n\times d}$ to the soft TCN assignment matrix $S\in {{\mathbb{R}}}^{n\times c}$, which can be formulated as below,

2 $$S={\rm{softmax}}\,\left(\text{linear}\left(X;{\Theta }_{3}\right)\right)$$

where each element in *S* represents the probability of a cell (row) belonging to one of the *c* TCNs (columns). *c* is a user-specified hyperparameter and represents the maximum number of TCNs to be detected. *Θ*_**3**_ is a trainable parameter matrix in this linear layer. Note that the optimal number of TCNs is automatically learned by this deep learning module and could be smaller than *c*. Next, we used the following MinCut-based loss function^14^ to optimize the matrix *S* in an unsupervised way,

3 $${L}_{\mathrm{MinCut}}=-\frac{\mathop{\sum }\nolimits_{j=1}^{c}{({S}^{T}AS)}_{{jj}}}{\mathop{\sum }\nolimits_{j=1}^{c}{({S}^{T}DS)}_{{jj}}}+{{\Big\Vert}\frac{S^{T}S}{{{||}{S}^{T}S{||}}_{F}}-\frac{{I}_{c}}{\sqrt{c}}{\Big\Vert}}_{F}$$

where $D\in {{\mathbb{R}}}^{n\times n}$ is a diagonal matrix in which each diagonal element is the sum of the corresponding row in the adjacency matrix *A*. The loss function *L*_MinCut_ is the sum of two terms. The left term is used to address the normalized MinCut problem in graph theory with the objective of partitioning the graph into *c* disjoint connected components with similar sizes by removing the minimum number of edges. The right term encourages the soft TCN assignment matrix *S* to be orthogonal to make the TCN membership of each node unambiguous. ‖∙‖_*F*_ denotes the Frobenius norm. This loss function can be used alone for an unsupervised learning task, that is, ${L}_{\mathrm{Unsup}}={L}_{\mathrm{MinCut}}$, to identify TCNs for single-cell spatial omics maps individually (Fig. 1b).

The second component of CytoCommunity is used to obtain a robust graph partitioning as the final TCNs by conducting ensemble learning (Fig. 1a, bottom). Specifically, the soft TCN assignment module in the first component is run multiple times to generate multiple learned matrices *S*. For each of them, the hard assignment is performed by assigning the cell (row) to the TCN (column) with the highest probability. Then, we used the majority vote strategy to conduct an ensemble procedure on those hard TCN assignments to determine the final set of TCNs. To demonstrate the utility of this ensemble approach, we performed a robustness experiment on the challenging MERFISH dataset with multiple small TCNs (Extended Data Fig. 2). We ran CytoCommunity with a different number of models to be learned by the first component. We then conducted comparisons of the TCN partitions generated by replicate experiments and found that the ensemble procedure improved the robustness of the result when the number of models increased. Our results showed that the ensemble procedure based on 20 models is sufficient to obtain a stable TCN partition.

### Supervised model for de novo identification of condition-specific TCNs

Given a dataset of multiple spatial omics maps from different conditions, TCNs can be identified for each spatial map first and then aligned across different maps to identify condition-specific TCNs. However, TCN alignment is analogous to community alignment in graphs, which is NP-hard^47^. To tackle this problem, we used differentiable graph pooling to generate an embedding representation of the whole graph that preserves the TCN partition information. By adapting the unsupervised graph partitioning model described above to a graph pooling-based graph classification framework, TCNs in different spatial maps are automatically aligned during soft TCN assignment learning, facilitating de novo identification of condition-specific TCNs (Fig. 1a, top). Specifically, after obtaining the soft TCN assignment matrix $S\in {{\mathbb{R}}}^{n\times c}$ using graph convolution and fully connected layers, we additionally used a differentiable graph pooling layer^14,36^ formulated as equations (4) and (5) to generate a coarsened graph of the original cellular spatial graph with the adjacency matrix $A^{{{\mathrm{Pooled}}}}\in {{\mathbb{R}}}^{c\times c}$ and a matrix of pooled node embeddings $X^{{\mathrm{Pooled}}}\in {{\mathbb{R}}}^{c\times d}$. Note that this coarsened graph is a fully connected graph with each pooled node corresponding to a TCN that includes a group of nodes (cells) with similar soft TCN assignments; the edge weights represent the connectivity strength between TCNs. For example, the coarsened graph in Fig. 1a consists of four pooled nodes, each of which has the same color with the TCN to be detected in the original cellular spatial graph:

4 $$X^{{\mathrm{Pooled}}}=S^TX$$

5 $$A^{{\mathrm{Pooled}}}=S^TAS$$

Then, we used *X*^**Pooled**^ and *A*^**Pooled**^ as inputs to another graph convolution layer similar to that described in equation (1) to integrate the pooled node features and their local neighborhood information in the coarsened graph, generating an updated embedding vector for each pooled node. The average of these new embedding vectors of the pooled nodes is an embedding vector of the whole graph, which is in turn used as the input to a graph classifier implemented by two fully connected layers with the softmax activation function. The overall supervised loss function is defined as:

6 $${L}_{\mathrm{Sup}}={\beta \times L}_{\mathrm{MinCut}}+(1-\beta )\times {L}_{\mathrm{{CE}}}$$

where *β* is a weight parameter to balance the *L*_MinCut_ loss used for graph partitioning and the cross-entropy *L*_CE_ loss used for graph classification. Trained with the joint loss function, this model can directly learn condition-specific TCNs under the supervision of sample labels (Fig. 1c).

For the triple-negative breast cancer MIBI-TOF, CRC CODEX and breast cancer IMC datasets in this study, we performed ten sets of tenfold cross-validation to evaluate the prediction performance of the model and used 100 optimal soft TCN assignment matrices generated during the cross-validation to conduct the TCN ensemble procedure for robust TCN identification. Note that the supervision signals are disease-relevant labels (for example, low-risk or high-risk), which are independent of patient-level information. For each of the three datasets, the training and test datasets were split based on the principle of tenfold cross-validation; the training samples were input to the model in a randomized fashion with no patient order or any other patient information. Therefore, during the training process, the model does not know which images come from the same patient. The model only knows about the class label (for example, low-risk or high-risk) of each image during training, which prevents data leakage. We also empirically set *β* to 0.9 because of our emphasis on graph partitioning and set the maximum number of TCNs to be identified to ten.

Unlike the unsupervised version, supervised CytoCommunity requires users to tune two training hyperparameters, that is, the size of a mini-batch and the learning rate. The mini-batch size is commonly set to the power of 2 because of efficiency. We used the mini-batch sizes of 16, 64 and 32 for the triple-negative breast cancer MIBI-TOF, CRC CODEX and breast cancer IMC datasets, respectively. These numbers are closest to half the sizes of the training sets for the three datasets, which can be considered as the suggested default value for mini-batch size using supervised CytoCommunity. For the learning rate, we adopted a commonly used strategy that the learning rate should be increased as the mini-batch size increases. Thus, we set the learning rates to 1.0 × 10^−4^, 1.0 × 10^−^^3^ and 1.0 × 10^−^^3^ for the three datasets, respectively.

### Running of published methods

We compared the performance of CytoCommunity with six other spatial domain detection methods, including five unsupervised methods, Spatial-LDA^9^, UTAG^10^, STAGATE^8^, BayesSpace^5^ and stLearn^6^, and one supervised method SPACE-GM^11^ (Supplementary Tables 2 and 3). As required by these methods, cell-type annotation and cell spatial coordinates were used as inputs to Spatial-LDA and SPACE-GM, while protein or mRNA expression data and cell spatial coordinates were used as inputs to the other four methods. For benchmarking purposes, the number of TCNs to be detected were adjusted to be consistent with the manual annotation from the original studies^16,17,48–50^ as much as possible by tuning the hyperparameters of the compared methods.

The Python package spatial-lda (v.0.1.3) was applied to four datasets (Supplementary Table 1). By considering all cells as index cells, we first used the featurize_samples and make_merged_difference_matrices functions for image featurization. Then, TCNs were detected using the spatial_lda.model.train function. For the mouse spleen CODEX dataset, the parameters were set to be max_dirichlet_iter = 30 and max_dirichlet_ls_iter = 30. Parameters were set as default for the other datasets. Note that the number of TCNs identified by this method may be fewer than the prespecified number.

The Python package STAGATE-pyG (v.1.0.0) was applied to five datasets (Supplementary Table 1). For each spatial omics map, a cell spatial neighbor network was constructed using the Cal_Spatial_Net and Stats_Spatial_Net functions. The train_STAGATE function was then used to learn low-dimensional latent representations of cells, which were considered as inputs to the Louvain clustering algorithm for TCN detection. The scanpy.pp.neighbors and scanpy.tl.louvain functions were used with resolution = 0.25 for all three images in the mouse spleen CODEX dataset. For the mouse hypothalamic preoptic region MERFISH dataset, the parameter resolution was set to 0.5, 0.45, 0.6, 0.62 and 0.76 for image Bregma −0.14, Bregma −0.04, Bregma +0.06, Bregma +0.16 and Bregma +0.26, respectively. For the other datasets, the parameters were set based on the official tutorial at https://stagate.readthedocs.io/en/latest/index.html.

The R package BayesSpace (v.1.5.1) was applied to five datasets (Supplementary Table 1). For the mouse spleen CODEX dataset, the top 15 principal components were considered and all 30 protein markers were used as highly variable genes (n.HVGs) in the preprocessing function spatialPreprocess. TCNs were identified using the spatialCluster function with nrep = 5,000 and burn.in = 100. For the mouse hypothalamic preoptic region MERFISH and visual cortex STARmap datasets, the parameter n.HVGs was set to 155 and 1,020, respectively. For the other datasets, default parameters were used.

The Python package stlearn (v.0.4.0) was applied to five datasets (Supplementary Table 1). TCNs were identified using the stlearn.tl.clustering.louvain function with resolution = 0.25 for all three images in the mouse spleen CODEX dataset. For the MERFISH dataset, the parameter resolution was set to 0.35, 0.5, 0.8, 0.9 and 1.3 for image Bregma −0.14, Bregma −0.04, Bregma +0.06, Bregma +0.16 and Bregma +0.26, respectively. For the STARmap dataset, the parameter resolution was set to 1.5. For the other datasets, parameters were set based on the official tutorial at https://stlearn.readthedocs.io/en/latest/tutorials.html.

The Python package UTAG (v.0.1.1) was applied to six datasets (Supplementary Table 1). For the mouse spleen CODEX dataset, the parameter max_dist was set to 100 for all three images and the parameter resolution was set to 0.05, 0.06 and 0.03 for the BALB/c-1, BALB/c-2 and BALB/c-3 images, respectively. For the mouse hypothalamic preoptic region MERFISH dataset, the parameter max_dist was set to 50 for all five images and the parameter resolution was set to 0.2, 0.2, 0.29, 0.4 and 0.5 for image Bregma −0.14, Bregma −0.04, Bregma +0.06, Bregma +0.16 and Bregma +0.26, respectively. For the mouse visual cortex STARmap dataset, the parameter max_dist was set to 10 and the parameter resolution was set to 0.4. For the triple-negative breast cancer MIBI-TOF dataset, the parameter max_dist was set to 60 and the parameter resolution was set to 0.05 for all images in the dataset. For the pancreatic ductal adenocarcinoma (PDAC) spatial transcriptome (ST) dataset, the parameter max_dist was set to 20 and the parameter resolution was set to 0.07. For the dorsolateral prefrontal cortex (DLPFC) Visium dataset, the parameter max_dist was set to 20 and the parameter resolution was set to 0.1.

The Python code of SPACE-GM (v.0.1.2) was downloaded from https://gitlab.com/enable-medicine-public/space-gm. This code was applied to three datasets with sample labels (Supplementary Table 1) based on the official tutorial. We observed normally decreasing training losses for all three datasets. To evaluate prediction performance, we conducted ten sets of tenfold cross-validation using all three datasets as for the supervised CytoCommunity. To evaluate TCN identification performance, we only applied SPACE-GM to the triple-negative breast cancer MIBI-TOF dataset because of the feasibility of quantitative evaluation of TCNs in this dataset. We first split the dataset into training and testing datasets using a tenfold cross-validation. We then used the get_random_sampled_subgraphs function to randomly sample 100,000 subgraphs from the training dataset as the reference dataset based on the recommendation in the original study^11^. Next, we used the get_embedding function to generate embeddings of those reference subgraphs, which were used for fitting a dimension reduction model and a *k*-means clustering model. Finally, these fitted models were applied to the testing dataset to generate TCN partitions of each image.

### Quantitative performance evaluation using the CODEX, MERFISH, STARmap, ST and Visium datasets

We used two metrics, the macro-F1 and AMI scores to quantitatively evaluate the performance of six compared methods. For the mouse spleen CODEX data, the GT assignment of cells to four known splenic compartments, that is, red pulp, marginal zone, B cell zone and PALS, were obtained from the authors of the original study^16^. For the mouse hypothalamus MERFISH data, the GT outlines of the nuclei regions were obtained from the original study^17^. The nucleus membership of cells was manually assigned by overlaying the outlines of nuclei and the MERFISH images. For the mouse visual cortex STARmap data, cortical layer annotations were obtained from the original study^48^. For the ST and Visium data, spatial domain annotations were obtained from the original studies^49,50^. For each sample, the macro-F1 and AMI scores are defined as follows and as computed using the Python package scikit-learn (v.1.2.2):

7 $$\mathrm{F1{score}}=\frac{2\times ({\mathrm{precision}}\times {\mathrm{recall}})}{{\mathrm{precision}+{{\mathrm{recall}}}}}$$

8 $${\mathrm{Precision}}=\frac{\mathrm{TP}}{{\mathrm{TP}}+{\mathrm{FP}}}$$

9 $${\mathrm{Recall}}=\frac{{\mathrm{TP}}}{{\mathrm{TP}}+{\mathrm{FN}}}$$

where the F1 score is computed based on the true positives (TPs), false positives (FPs) and false negatives (FNs) of the TCN predictions compared to the GT assignment of cells. The macro-F1 score was defined as the average F1 score across all GT TCN types in a dataset. AMI measures the agreement between predicted and GT TCNs using the Shannon information theory^51^:

10 $${\mathrm{AMI}}=\frac{I\left({\mathrm{GT}}{\rm{;}}{\mathrm{TCN}}\right)-E\{I\left({\mathrm{GT}}{\rm{;}}{\mathrm{TCN}}\right)\}}{\frac{1}{2}\left[H\left({\mathrm{GT}}\right)+H\left({\mathrm{TCN}}\right)\right]-E\{I\left({\mathrm{GT}}{\rm{;}}{\mathrm{TCN}}\right)\}}$$

where $E\{I\left({\mathrm{GT};\mathrm{TCN}}\right)\}$ represents the expected mutual information between the GT and predicted TCN labels of cells. *H*(GT) and *H*(TCN) are the entropy of the GT and predicted TCN labels, respectively. Both macro-F1 and AMI take into account unbalanced classes in the data (for example, TCNs with different numbers of cells).

### Cell-type enrichment score in TCNs

To quantitatively measure the composition of cell types in the identified TCNs, we defined an enrichment score of each cell type in each TCN as −log_10_(*P*). The *P* value was computed using a hypergeometric test based on the following four numbers: (1) the number of cells of a given type in the TCN; (2) the total number of cells in the TCN; (3) the number of cells of the given type in the single-cell spatial map; and (4) the total number of cells in the spatial map. *P* values were adjusted for multiple testing using the Benjamini–Hochberg method^45^.

### Analysis of the cell–cell communication pattern

Considering the skewed distribution of the cell-type enrichment scores (Supplementary Fig. 5a,e), we chose to compute the Spearman rank correlation coefficient because it is more robust than the Pearson correlation coefficient^52^ in identifying cell-type communication patterns using cell-type enrichment scores. By comparison (Supplementary Fig. 5b–d,f–h), we found that a CLR-specific cell–cell communication pattern (CD68^+^CD163^+^ macrophages and plasma cells) in CRC cannot be revealed if the Pearson correlation coefficient is used (Supplementary Fig. 5c).

To identify the associations among cell types located in different TCNs, we conducted canonical correlation analysis (CCA) of each TCN pair using the cell-type enrichment scores. For each TCN, we selected the five most enriched cell types based on the average enrichment scores across patient samples as the observed variables of the TCN. Then, the canonical correlation model between each TCN pair was constructed using the cc function of the R package CCA (v.1.2.1). We computed the *P* values of the canonical correlation coefficients using the permutation test-based p.perm function from the R package CCP (v.1.2). To facilitate interpretation of the CCA results, we further investigated the correlations between the dominant cell types identified based on their normalized weights in the first canonical variate pair to describe the cell–cell communication patterns between TCNs.

### Survival analysis

For the breast cancer IMC dataset^12^, we stratified patients into low-risk and high-risk groups based on the median OS using only 79 deceased patients. We did not consider censored patients because their OS time is unknown.

To further evaluate the prognosis ability of the TCNs identified in high-risk patients with breast cancer, we used the TCN-based cell-type enrichment scores as patient features to perform *k*-means clustering and identified three patient subgroups, which we named TCN-induced subgroups 1, 2 and 3 (Extended Data Fig. 10). Subgroup 3 contained fewer than three patients and was thus removed from the survival analysis because of their effect on statistical power and to be consistent with the criteria proposed by original study. For comparison, we also downloaded the single-cell pathology (SCP) subtyping annotation from the original study and identified 17 SCP subgroups among high-risk patients with cancer. Two SCP subgroups with more than three patients were used for the survival analysis. All Kaplan–Meier survival curves and corresponding log-rank test *P* values were computed using the R package survival (v.3.2-13).

### Reporting summary

Further information on research design is available in the Nature Portfolio Reporting Summary linked to this article.

## Online content

Any methods, additional references, Nature Portfolio reporting summaries, source data, extended data, supplementary information, acknowledgements, peer review information; details of author contributions and competing interests; and statements of data and code availability are available at 10.1038/s41592-023-02124-2.

### Supplementary Information

Supplementary Information Supplementary Notes, Figs. 1–8, Tables 1–3 and references.

Reporting Summary

## Data Availability

This study used eight publicly available datasets (Supplementary Table 1), including a mouse spleen CODEX dataset (https://data.mendeley.com/datasets/zjnpwh8m5b/1), a mouse hypothalamic preoptic region MERFISH dataset (https://datadryad.org/stash/dataset/doi:10.5061/dryad.8t8s248), a mouse visual cortex STARmap dataset (http://clarityresourcecenter.org/), a human triple-negative breast cancer MIBI-TOF dataset (https://mibi-share.ionpath.com), a human CRC CODEX dataset (https://data.mendeley.com/datasets/mpjzbtfgfr/1), a human breast cancer IMC dataset (https://zenodo.org/record/3518284#.Y2UQ0-xBybg), a human PDAC ST dataset (GSE111672) and a human DLPFC Visium dataset (http://research.libd.org/spatialLIBD/).

## Code availability

The software package implementing the CytoCommunity algorithm has been deposited at GitHub (https://github.com/tanlabcode/CytoCommunity) and Zenodo (https://www.zenodo.org/record/8335454)^53^.
