# Supplementary material for: Unsupervised and supervised discovery of tissue cellular neighborhoods from cell phenotypes
Source: Nat Methods. 2024 Jan 8;21(2):267–78. doi: 10.1038/s41592-023-02124-2 (PMC10864185; doi:10.1038/s41592-023-02124-2)
Supplement: Supplementary file 1 — Supplementary Notes, Figs. 1–8, Tables 1–3 and references. [file 41592_2023_2124_MOESM1_ESM.pdf]

nature methods

Article

https://doi.org/10.1038/s41592-023-02124-2

Unsupervised and supervised discovery of tissue cellular neighborhoods from cell phenotypes

In the format provided by the authors and unedited

Table of Contents

Supplementary Notes···2

Supplementary Figures···3

Supplementary Tables··11

Supplementary References ···15

Supplementary Notes

Performance evaluation using the STARmap data

To further demonstrate the general applicability of CytoCommunity, we applied it to a second single-cell spatial transcriptomics dataset of healthy mouse visual cortex generated using the Spatially-resolved Transcript Amplicon Readout mapping (STARmap) technology [1] (Supplementary Table 1). The dataset consists of expression measures of 1,020 gene across 1,207 cells covering 15 cell types (Supplementary Fig. 6a). The cells are spatially distributed in seven separate cortical layers manually annotated by the authors [1] (Supplementary Fig. 6b). Here we regarded these cortical layers as ground-truth TCNs. It is worth noting that most of the 15 cell types are located in multiple cortical layers. CytoCommunity is ranked first and second among all compared methods in terms of AMI and macro-F1 scores, respectively (Supplementary Fig. 6d). We also found that TCNs identified by CytoCommunity have more clear boundaries than the other methods (Supplementary Fig. 6c). Although STAGATE has slightly higher macro-F1 score than CytoCommunity, it cannot distinguish the HPC layer (red) from the L5 layer (green) while CytoCommunity can (Supplementary Fig. 6c).

Performance evaluation using spot-based spatial transcriptomics data

To extend the applicability of CytoCommunity to spatial transcriptomics data with spot resolution, we first used a state-of-the-art cell-type deconvolution method, CARD [2], to estimate the cell type compositions at each spot. Then, a spot-spot proximity graph with inferred cell type fractions as node attributes was constructed as the input to CytoCommunity. Given a spatial transcriptomics dataset, CARD requires a matched single-cell RNA-sequencing (scRNA-seq) dataset measured from the same tissue as the reference for cell-type deconvolution. CARDfree [2] is a reference-free variant of CARD and only needs cell-type markers for deconvolution. We applied both CARD and CARDfree together with CytoCommunity to two spatial transcriptomics datasets of human pancreatic ductal adenocarcinoma (PDAC) [3] (Supplementary Fig. 7a) and dorsolateral prefrontal cortex (DLPFC) [4] (Supplementary Fig. 8a), respectively. When analyzing the PDAC dataset, we used both CARD and CARDfree to estimate cell type fractions at each spot (Supplementary Fig. 7b). The reference scRNA-seq data from [3] were used for CARD and gene markers of 20 cell types of PDAC from [2] were used for CARDfree. When analyzing the DLPFC dataset, we only used CARDfree for cell-type deconvolution (Supplementary Fig. 8b) based on gene markers of 23 cell types of the brain [5].

We found that CytoCommunity successfully identified TCNs of irregular shapes in PDAC (Supplementary Fig. 7c) and layered TCNs in DLPFC (Supplementary Fig. 8c). It is worth noting that most deconvolution methods require scRNA-seq data as the reference. However, CytoCommunity has comparable performance with the state-of-the-art methods when used together with reference-free deconvolution methods such as CARDfree [2] (Supplementary Figs. 7d and 8d). Taken together, these results suggest that CytoCommunity is applicable to spot-based spatial transcriptomics data without additional input by leveraging cell-type deconvolution methods such as CARDfree.

Supplementary Figures

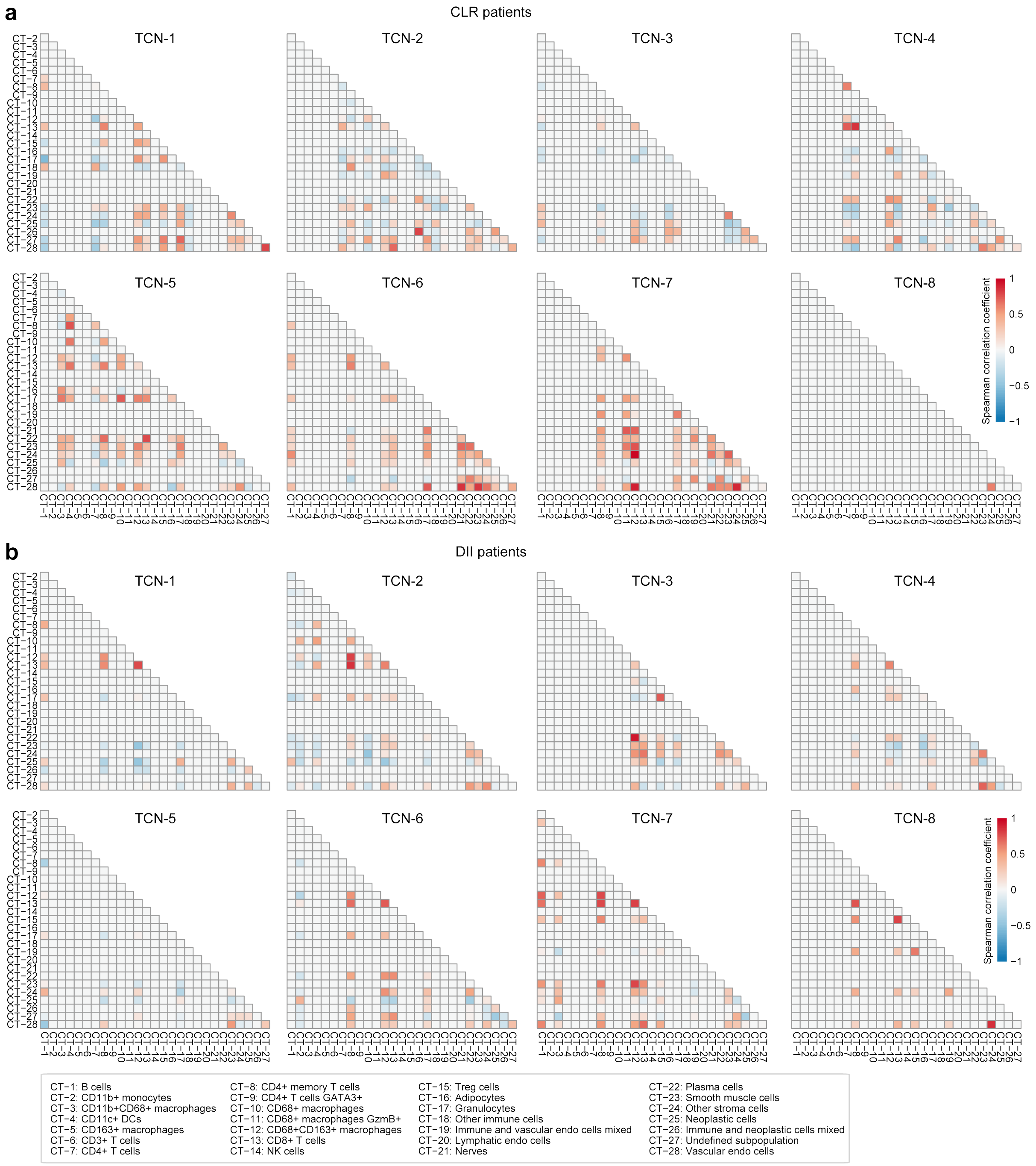

Supplementary Fig. 1. Cell type associations within TCNs in colorectal cancer. Heatmaps for Spearman's rank correlations of the enrichment scores of any two cell types (CTs) within each of the 8 TCNs identified in CLR **(a)** and DII **(b)** patients.

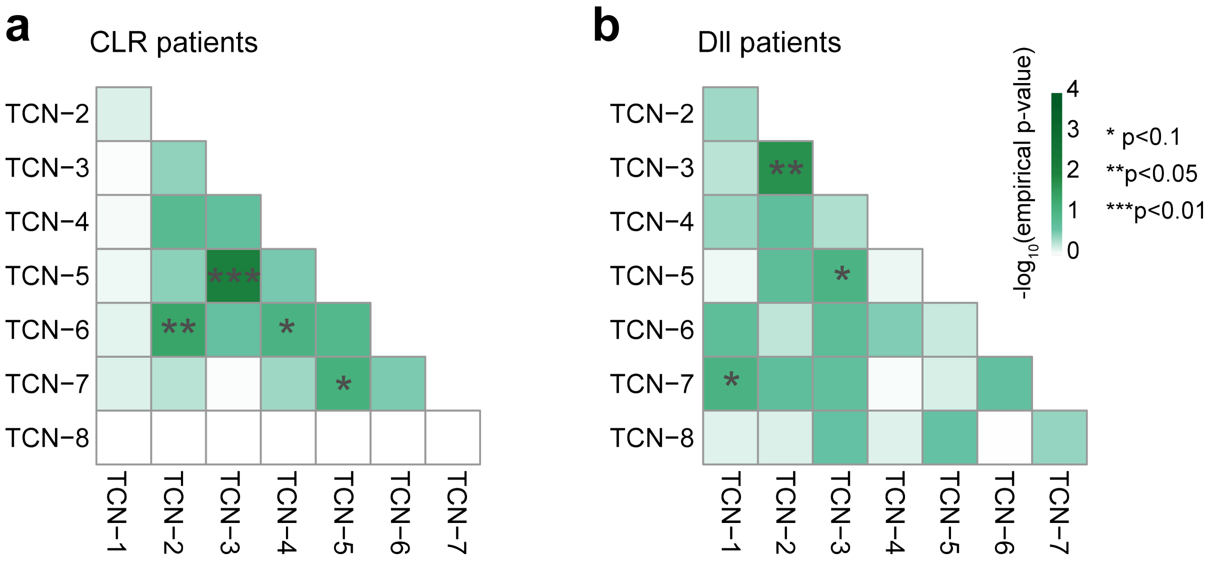

Supplementary Fig. 2. Cell type associations between TCNs in colorectal cancer. Heatmaps for empirical p-values of canonical correlation coefficients of the cell type enrichment scores of TCN pairs in CLR **(a)** and DII **(b)** patient groups. *P*-values were computed using one-sided permutation test-based *p.perm* function from the R package "CCP (v1.2)".

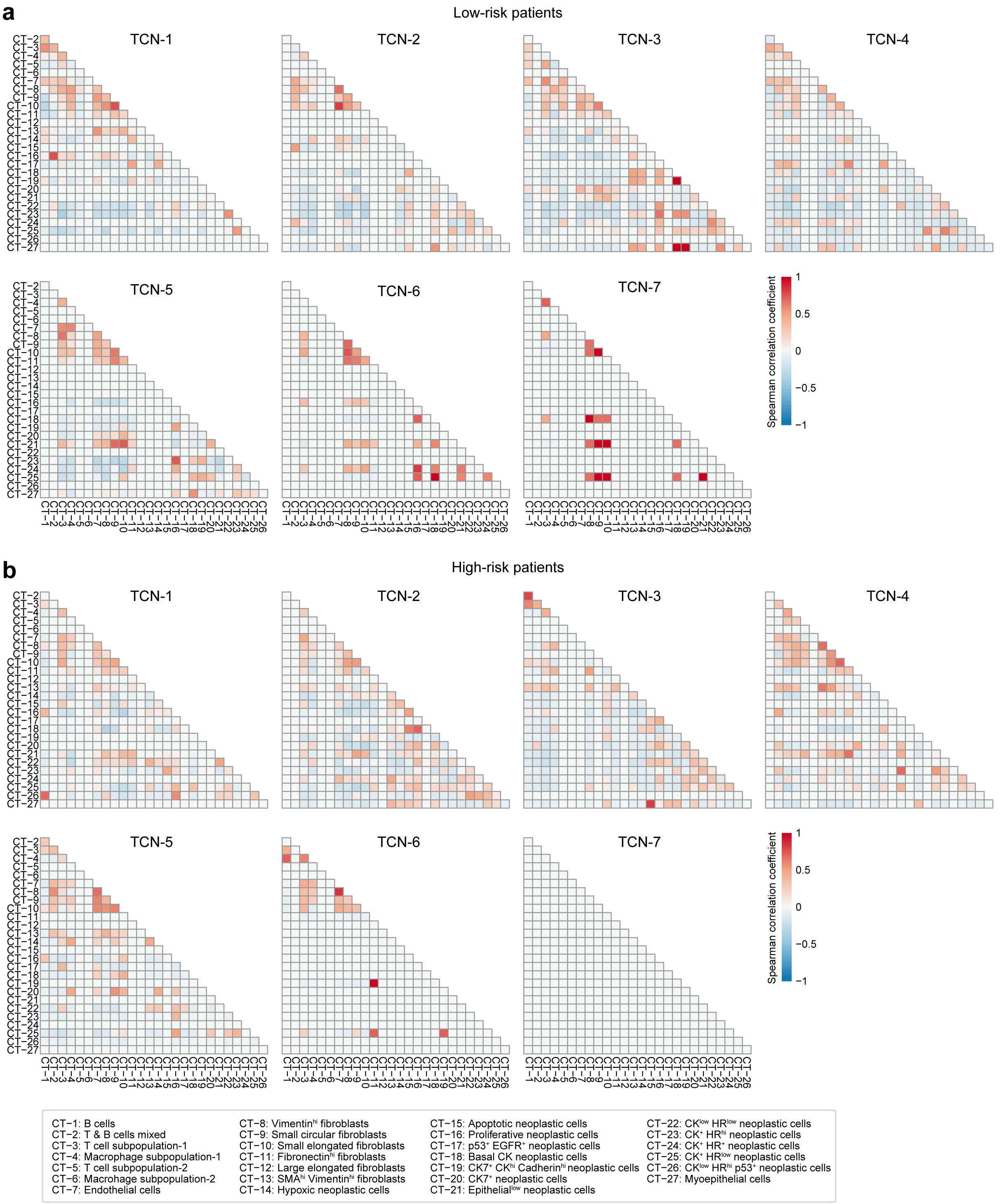

Supplementary Fig. 3. Cell type associations within TCNs in breast cancer. Heatmaps for correlations of the enrichment scores of any two cell types (CTs) within each of the seven TCNs identified in low-risk **(a)** and high-risk **(b)** patients.

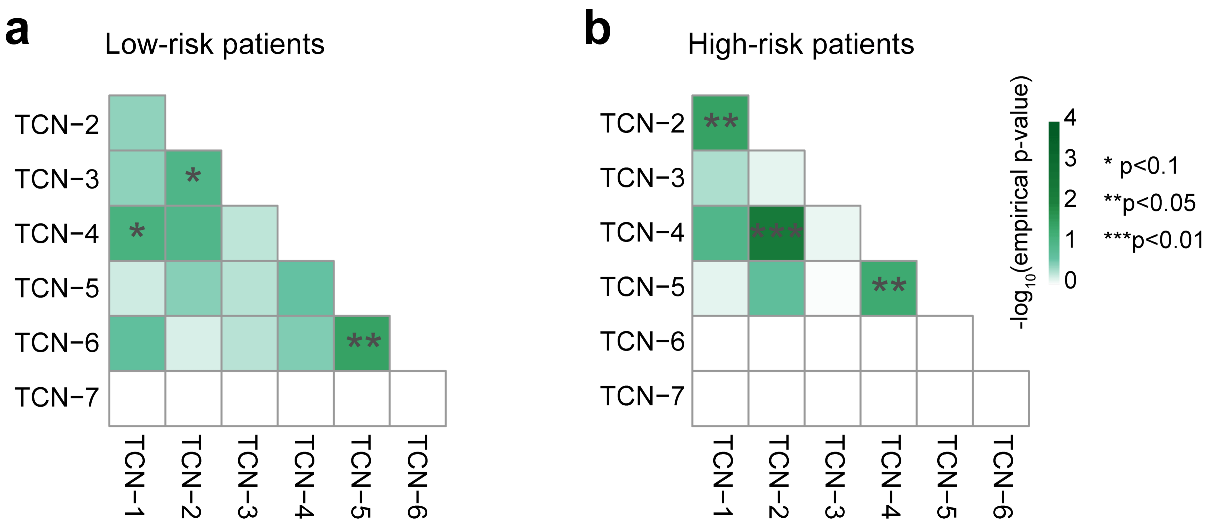

Supplementary Fig. 4. Cell type associations between TCNs in breast cancer. Heatmaps for empirical p-values of canonical correlation coefficients of the cell type enrichment scores of TCN pairs in low-risk **(a)** and high-risk **(b)** patient groups. *P*-values were computed using one-sided permutation test-based *p.perm* function from the R package "CCP (v1.2)".

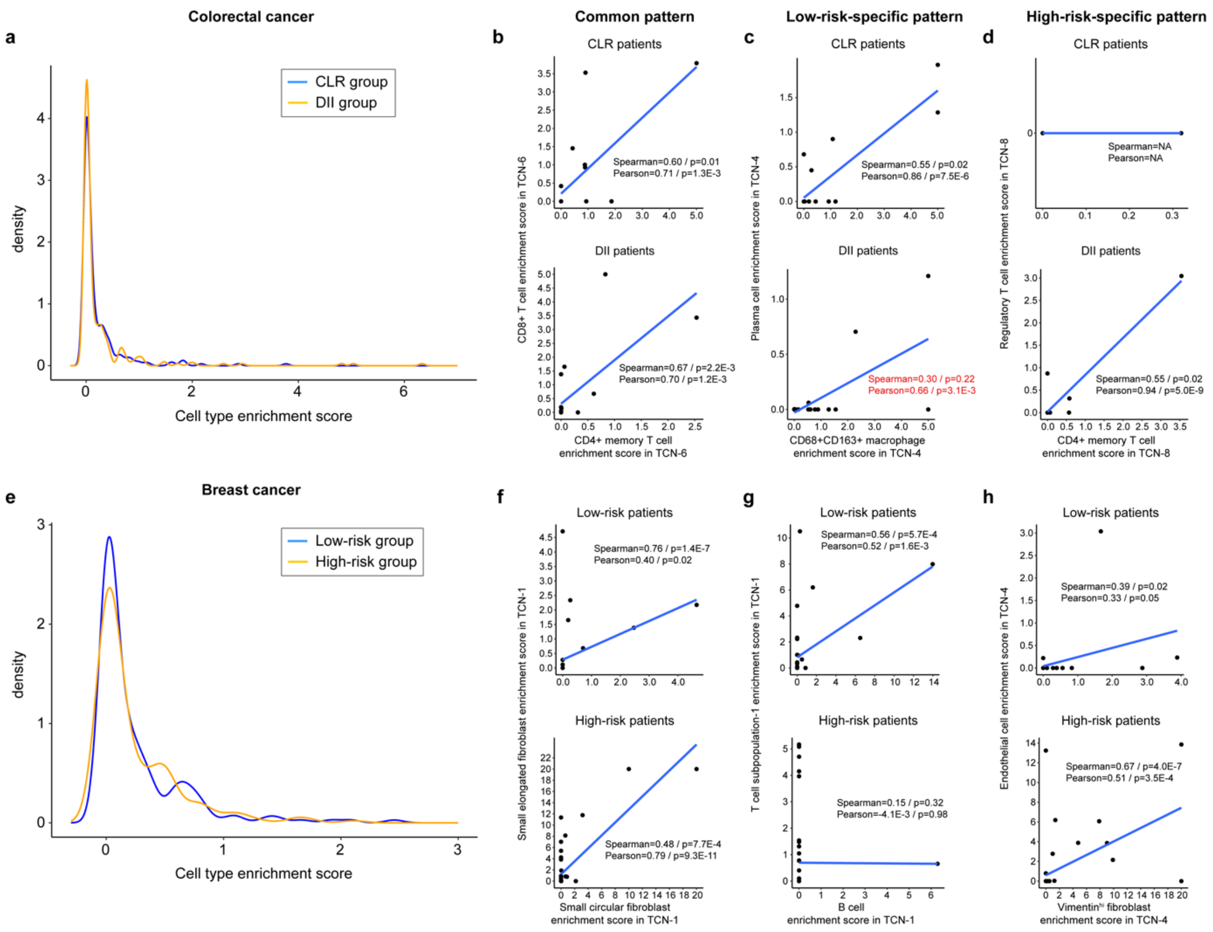

Supplementary Fig. 5. Justification for using Spearman's rank correlation coefficient in cell-cell communication pattern analysis. (a and e) Skewed distributions of cell type enrichment scores of colorectal cancer CODEX dataset and breast cancer IMC dataset, respectively. **(b-d)** Common, CLR- and DII-specific cell-cell communication patterns in colorectal cancer, same examples as Fig. 5d-f. **(f-h)** Common, low-risk- and high-risk-specific cell-cell communication patterns in breast cancer, same examples as Fig. 6e-g. Spearman's rank correlation and Pearson correlation coefficients of the enrichment scores of two indicated cell types as well as corresponding two-sided Spearman's rho test and *t*-test *p*-values were shown. A cell-cell communication pattern with different correlation significance was highlighted in red.

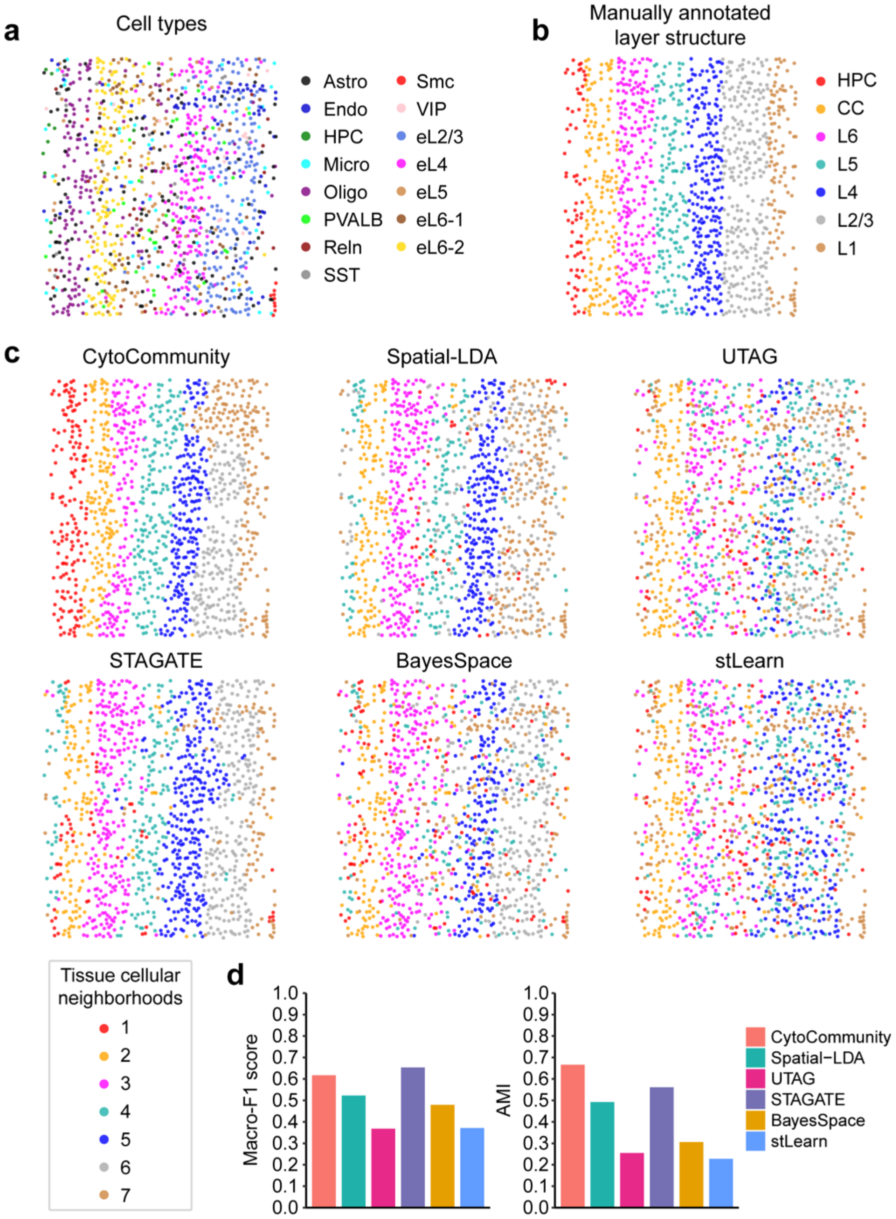

Supplementary Fig. 6. Performance evaluation of CytoCommunity using mouse visual cortex STARmap data. (a, b) Single-cell spatial map of healthy mouse visual cortex sample. Cells are colored based on cell type annotation (a) or manually annotated cortical layer structure (b) from the original study [1]. **(c)** TCNs identified by the compared methods. **(d)** Barplots of Macro-F1 scores and Adjusted Mutual Information (AMI) scores computed by comparing detected TCNs with manually annotated layer structures.

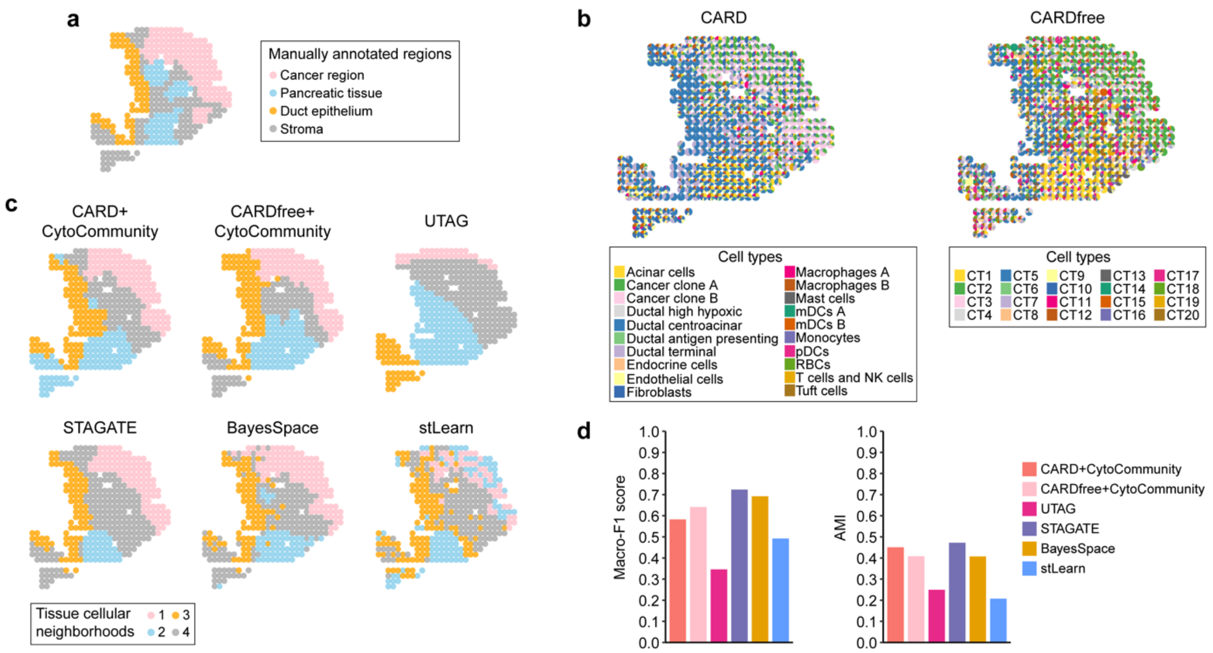

Supplementary Fig. 7. Performance evaluation of CytoCommunity using human pancreatic ductal adenocarcinoma (PDAC) spatial transcriptomic data with spot resolution. **(a)** The tissue domains of the PDAC sample [3] manually annotated by the study [2] are considered as the ground truth for TCN identification. **(b)** Spatial pie chart plots showing the inferred cell type composition of each spot by the cell-type deconvolution method CARD [2] and CARDfree [2], respectively. CARDfree is a variant of CARD without using single-cell RNA-seq reference data. mDCs, myeloid dendritic cells; pDCs, plasmacytoid dendritic cells; RBCs, red blood cells; NK cells, natural killer cells. **(c)** TCNs identified by CARD+CytoCommunity, CARDfree+CytoCommunity, UTAG, STAGATE, BayesSpace and stLearn, respectively. **(d)** Barplots of Macro-F1 scores and Adjusted Mutual Information (AMI) scores computed by comparing detected TCNs with manually annotated domains.

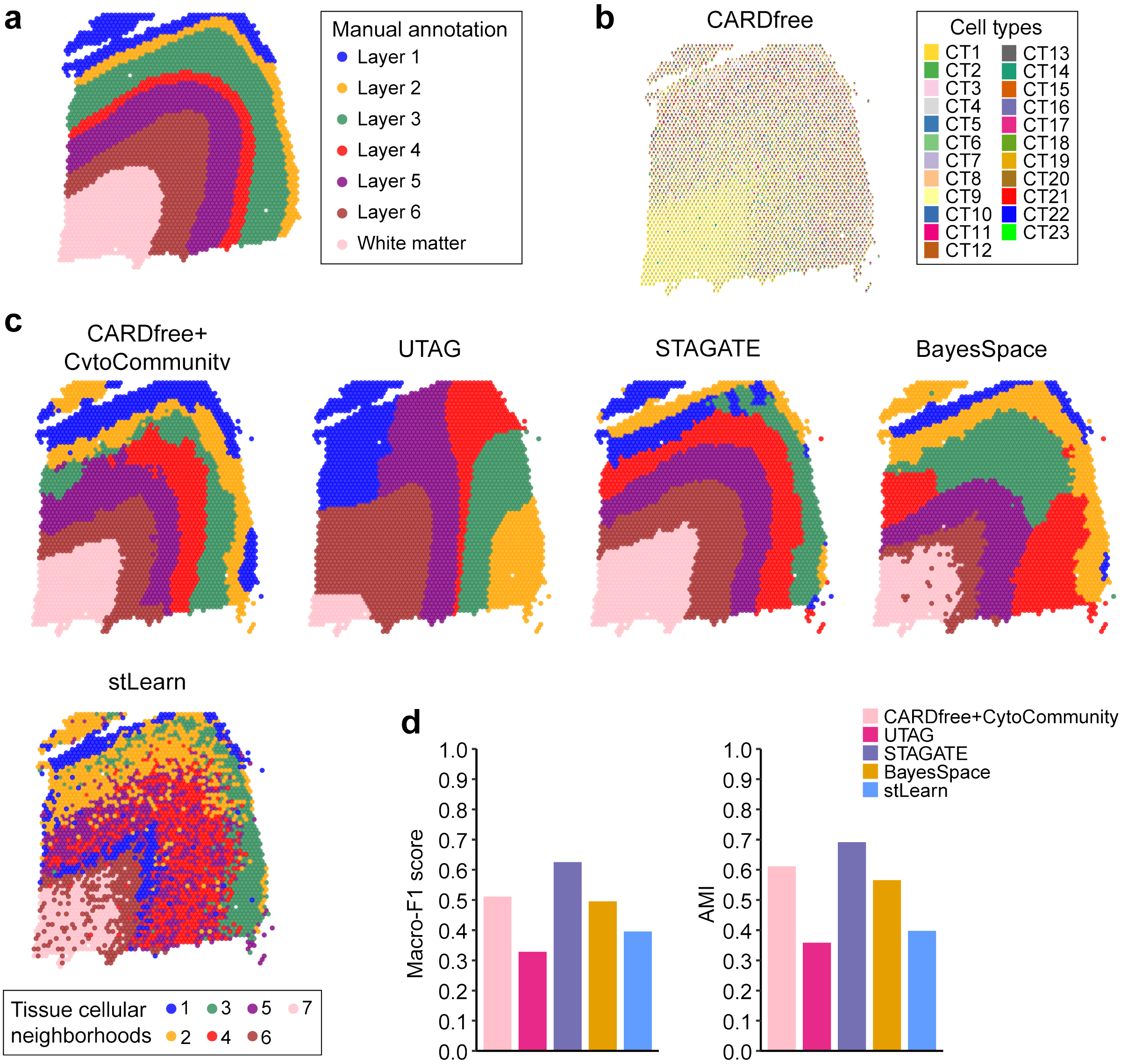

Supplementary Fig. 8. Performance evaluation of CytoCommunity using human dorso-lateral prefrontal cortex (DLPFC) spatial transcriptomics data with spot resolution. (a) Manually annotated six cortical layers and white matter in a tissue sample "151676" of DLPFC from the original study [4] are considered as the ground truth for TCN identification. **(b)** Spatial pie chart plot showing the inferred cell type composition of each spot by the cell-type deconvolution method CARDfree [2] based on gene markers of 23 cell types in the brain [5]. CARDfree is a variant of CARD [2] without using single-cell RNA-seq reference data. **(c)** TCNs identified by CARDfree+CytoCommunity, UTAG, STAGATE, BayesSpace and stLearn, respectively. **(d)** Barplots of Macro-F1 scores and Adjusted Mutual Information (AMI) scores computed by comparing detected TCNs with manually annotated cortical domains.

Supplementary Tables

Supplementary Table 1. Summary of datasets used in this study.

Tissue	Technology	Resolution	Accession number/Website link	PubMed ID	Related figure	Applied method
Mouse spleen	CODEX	Single-cell	https://data.mendeley.com/datasets/zjnpwh8m5b/1	30078711	Figure 2	(1) Unsupervised CytoCommunity (2) Spatial-LDA (3) UTAG (4) STAGATE (5) BayesSpace (6) stLearn
Mouse hypothalamic preoptic region	MERFISH	Single-cell	https://datadryad.org/stash/dataset/doi:10.5061/dryad.8t8s248	30385464	Figure 3; Extended Data Figures 1-3	(1) Unsupervised CytoCommunity (2) Spatial-LDA (3) UTAG (4) STAGATE (5) BayesSpace (6) stLearn
Mouse visual cortex	STARmap	Single-cell	http://clarityresourcecenter.org/	29930089	Supplementary Figure 6	(1) Unsupervised CytoCommunity (2) Spatial-LDA (3) UTAG (4) STAGATE (5) BayesSpace (6) stLearn
Human triple-negative breast tumor	MIBI-TOF	Single-cell	https://mibi-share.ionpath.com	30193111	Figure 4; Extended Data Figures 1-2 and 4-5	(1) Supervised CytoCommunity (2) SPACE-GM (3) Unsupervised CytoCommunity (4) Spatial-LDA (5) UTAG
Human colorectal tumor	CODEX	Single-cell	https://data.mendeley.com/datasets/mpjzbtfgfr/1	32763154	Figure 5; Extended Data Figures 6-7; Supplementary Figures 1-2 and 5	(1) Supervised CytoCommunity (2) SPACE-GM
Human breast tumor	IMC	Single-cell	https://zenodo.org/record/3518284#.Y2UQ0-xBybg	31959985	Figure 6; Extended Data Figures 8-10; Supplementary Figures 3-5	(1) Supervised CytoCommunity (2) SPACE-GM

Human pancreatic ductal adenocarcinoma	ST	Spot	GSE111672	31932730	Supplementary Figure 7	(1) Unsupervised CytoCommunity<br>(2) UTAG<br>(3) STAGATE<br>(4) BayesSpace<br>(5) stLearn
Human dorsolateral prefrontal cortex	10x Genomics Visium	Spot	http://research.libd.org/spatialLIBD/	33558695	Supplementary Figure 8	(1) Unsupervised CytoCommunity<br>(2) UTAG<br>(3) STAGATE<br>(4) BayesSpace<br>(5) stLearn

Supplementary Table 2. Summary of features of methods compared in this study.

	**Input data resolution in original design**	**Learning mode**	**Input data features**	**Algorithmic features**
**CytoCommunity**	Single-cell	Supervised + Unsupervised	Cell type annotation, spatial coordinates	Graph neural network with MinCut loss, Differentiable graph pooling, Ensemble learning
**Spatial-LDA**	Single-cell	Unsupervised	Cell type annotation, spatial coordinates	Latent Dirichlet allocation
**UTAG**	Single-cell	Unsupervised	Protein/RNA expression, spatial coordinates	Message passing + Clustering
**STAGATE**	Spot	Unsupervised	Protein/RNA expression, spatial coordinates	Graph neural network + Clustering
**BayesSpace**	Spot	Unsupervised	Protein/RNA expression, spatial coordinates	Markov random field + Clustering
**stLearn**	Spot	Unsupervised	Protein/RNA expression, spatial coordinates	Convolutional neural network + Clustering
**SPACE-GM**	Single-cell	Supervised	Cell type annotation, spatial coordinates	Graph neural network + Clustering

Supplementary Table 3. Running time of compared methods in this study. All analyses were conducted on an Intel Xeon Gold-6226R 2.90 GHz CPU with 64 cores. Supervised methods were run with 10-fold cross-validation. Running time is recorded in minute.

	**Mouse visual cortex STARmap (1,207 cells)**	**Mouse hypothalamic preoptic region MERFISH (average 5,352 cells)**	**Mouse spleen CODEX (average 81,760 cells)**	**Human triple-negative breast cancer MIBI-TOF (34 images)**	**Human breast cancer IMC (83 images)**	**Human colorectal cancer CODEX (140 images)**
**Unsupervised CytoCommunity**	0.88	11.18	55.92	N/A	N/A	N/A
**Spatial-LDA**	4.03	27.02	161.58	N/A	N/A	N/A
**UTAG**	0.43	0.58	3.72	N/A	N/A	N/A
**STAGATE**	2.87	15.67	27.40	N/A	N/A	N/A
**BayesSpace**	8.40	26.77	66.97	N/A	N/A	N/A
**Supervised CytoCommunity**	N/A	N/A	N/A	53.10	52.54	53.27
**SPACE-GM**	N/A	N/A	N/A	441.25	473.95	615.18

Supplementary References

1. Wang, X. et al. Three-dimensional intact-tissue sequencing of single-cell transcriptional states. *Science* **361**, eaat5691 (2018).
2. Ma, Y. & Zhou, X. Spatially informed cell-type deconvolution for spatial transcriptomics. *Nature Biotechnology* **40**, 1349-1359 (2022).
3. Moncada, R. et al. Integrating microarray-based spatial transcriptomics and single-cell RNA-seq reveals tissue architecture in pancreatic ductal adenocarcinomas. *Nature Biotechnology* **38**, 333-342 (2020).
4. Maynard, K.R. et al. Transcriptome-scale spatial gene expression in the human dorsolateral prefrontal cortex. *Nature Neuroscience* **24**, 425-436 (2021).
5. Fischer, S. & Gillis, J. How many markers are needed to robustly determine a cell's type? *iScience* **24**, 103292 (2021).
