# Supplementary material for: Unsupervised and supervised discovery of tissue cellular neighborhoods from cell phenotypes
Source: Nat Methods. 2024 Jan 8;21(2):267–78. doi: 10.1038/s41592-023-02124-2 (PMC10864185; doi:10.1038/s41592-023-02124-2)
Supplement: Supplementary file 2 — Reporting Summary [file 41592_2023_2124_MOESM2_ESM.pdf]

nature portfolio

Corresponding author(s): Kai Tan, Yuxuan Hu

Last updated by author(s): Oct 26, 2023

Reporting Summary

Nature Portfolio wishes to improve the reproducibility of the work that we publish. This form provides structure for consistency and transparency in reporting. For further information on Nature Portfolio policies, see our Editorial Policies and the Editorial Policy Checklist.

Statistics

For all statistical analyses, confirm that the following items are present in the figure legend, table legend, main text, or Methods section.

n/a	Confirmed	
☐	☒	The exact sample size (*n*) for each experimental group/condition, given as a discrete number and unit of measurement
☐	☒	A statement on whether measurements were taken from distinct samples or whether the same sample was measured repeatedly
☐	☒	The statistical test(s) used AND whether they are one- or two-sided<br>*Only common tests should be described solely by name; describe more complex techniques in the Methods section.*
☐	☒	A description of all covariates tested
☐	☒	A description of any assumptions or corrections, such as tests of normality and adjustment for multiple comparisons
☐	☒	A full description of the statistical parameters including central tendency (e.g. means) or other basic estimates (e.g. regression coefficient) AND variation (e.g. standard deviation) or associated estimates of uncertainty (e.g. confidence intervals)
☐	☒	For null hypothesis testing, the test statistic (e.g. *F*, *t*, *r*) with confidence intervals, effect sizes, degrees of freedom and *P* value noted<br>*Give P values as exact values whenever suitable.*
☒	☐	For Bayesian analysis, information on the choice of priors and Markov chain Monte Carlo settings
☒	☐	For hierarchical and complex designs, identification of the appropriate level for tests and full reporting of outcomes
☐	☒	Estimates of effect sizes (e.g. Cohen's *d*, Pearson's *r*), indicating how they were calculated

Our web collection on statistics for biologists contains articles on many of the points above.

Software and code

Policy information about availability of computer code

Data collection	No software was used to collect data.
Data analysis	CytoCommunity (v1.0.0) proposed in this paper has been deposited at at GitHub (https://github.com/tanlabcode/CytoCommunity) and Zenodo (https://www.zenodo.org/record/8335454). Other compared methods are spatial-lda (v0.1.3), STAGATE-pyG (v1.0.0), BayesSpace (v1.5.1), stlearn (v0.4.0), UTAG (v0.1.1) and SPACE-GM (v0.1.2). Tools for data analysis include the Python package scikit-learn (v1.2.2), R packages CCA (v1.2.1), CCP (v1.2) and survival (v3.2-13).

For manuscripts utilizing custom algorithms or software that are central to the research but not yet described in published literature, software must be made available to editors and reviewers. We strongly encourage code deposition in a community repository (e.g. GitHub). See the Nature Portfolio guidelines for submitting code & software for further information.

Data

Policy information about availability of data

All manuscripts must include a data availability statement. This statement should provide the following information, where applicable:

- Accession codes, unique identifiers, or web links for publicly available datasets
- A description of any restrictions on data availability
- For clinical datasets or third party data, please ensure that the statement adheres to our policy

This study used eight publicly available datasets (Supplementary Table 1), including a mouse spleen CODEX dataset (https://data.mendeley.com/datasets/

zjnpwh8m5b/1), a mouse hypothalamic preoptic region MERFISH dataset (https://datadryad.org/stash/dataset/doi:10.5061/dryad.8t8s248), a mouse visual cortex STARmap dataset (http://clarityresourcecenter.org/), a human triple-negative breast cancer MIBI-TOF dataset (https://mibi-share.ionpath.com), a human colorectal cancer CODEX dataset (https://data.mendeley.com/datasets/mpjzbtfgfr/1), a human breast cancer IMC dataset (https://zenodo.org/record/3518284#.Y2UQ0-xBybg), a human pancreatic ductal adenocarcinoma ST dataset (GSE111672) and a human dorsolateral prefrontal cortex Visium dataset (http://research.libd.org/spatialLIBD/).

Human research participants

Policy information about studies involving human research participants and Sex and Gender in Research.

Reporting on sex and gender	N/A
Population characteristics	N/A
Recruitment	N/A
Ethics oversight	N/A

Note that full information on the approval of the study protocol must also be provided in the manuscript.

Field-specific reporting

Please select the one below that is the best fit for your research. If you are not sure, read the appropriate sections before making your selection.

☒ Life sciences ☐ Behavioural & social sciences ☐ Ecological, evolutionary & environmental sciences

For a reference copy of the document with all sections, see nature.com/documents/nr-reporting-summary-flat.pdf

Life sciences study design

All studies must disclose on these points even when the disclosure is negative.

Sample size	The mouse spleen CODEX dataset includes three samples with ground-truth annotation available. The mouse hypothalamic preoptic region MERFISH dataset includes five samples with ground-truth annotation available. The human triple-negative breast cancer MIBI-TOF dataset includes 15 compartmentalized and 19 mixed tumor samples, which constitute a stratified spatial omics dataset suitable for quantitative evaluation of supervised CytoCommunity. The human colorectal cancer CODEX dataset includes 68 and 72 samples from the CLR and DII patients, respectively, which constitute a risk-stratified spatial omics dataset suitable for testing supervised CytoCommunity. The human breast cancer IMC dataset includes 83 samples from deceased patients, which can be classified into two risk groups based on clinical information and are thus suitable for testing supervised CytoCommunity. The three samples respectively from the mouse visual cortex STARmap dataset, human pancreatic ductal adenocarcinoma ST dataset and human dorsolateral prefrontal cortex Visium dataset are all commonly used in performance evaluation of spatial domain detection methods and are thus used for testing unsupervised CytoCommunity.
Data exclusions	No data were excluded from the analyses.
Replication	Robustness score was used to verify the reproducibility of the study. All attempts at replication were successful. Specifically, the robustness evaluation on 1) the value of K in KNN graph, 2) the number of GNN models in the ensemble procedure and 3) granularity of cell type annotation are based on five, four and two independently performed experiments for each dataset, respectively.
Randomization	In the evaluation of supervised CytoCommunity and SPACE-GM, the training and test sets were split randomly based on the principle of 10-fold cross-validation.
Blinding	Blinding is not applicable to this study because our main finding is the development of a computational tool (CytoCommunity) and we used publicly available datasets for all data analysis.

Reporting for specific materials, systems and methods

We require information from authors about some types of materials, experimental systems and methods used in many studies. Here, indicate whether each material, system or method listed is relevant to your study. If you are not sure if a list item applies to your research, read the appropriate section before selecting a response.

Materials & experimental systems

n/a	Involved in the study
☒	☐ Antibodies
☒	☐ Eukaryotic cell lines
☒	☐ Palaeontology and archaeology
☒	☐ Animals and other organisms
☒	☐ Clinical data
☒	☐ Dual use research of concern

Methods

n/a	Involved in the study
☒	☐ ChIP-seq
☒	☐ Flow cytometry
☒	☐ MRI-based neuroimaging
